# Cofilin and Actin Dynamics: Multiple Modes of Regulation and Their Impacts in Neuronal Development and Degeneration

**DOI:** 10.3390/cells10102726

**Published:** 2021-10-12

**Authors:** James R. Bamburg, Laurie S. Minamide, O’Neil Wiggan, Lubna H. Tahtamouni, Thomas B. Kuhn

**Affiliations:** 1 Department of Biochemistry and Molecular Biology, Colorado State University, Fort Collins, CO 80523, USA; Laurie.Minamide@colostate.edu (L.S.M.); ONeil.Wiggan@ColoState.EDU (O.W.); Lubna.Tahtamouni@colostate.edu (L.H.T.); Tom.Kuhn@colostate.edu (T.B.K.); 2 Department of Biology and Biotechnology, The Hashemite University, Zarqa 13115, Jordan; 3 Department of Chemistry and Biochemistry, University of Alaska, Fairbanks, AK 99775, USA

**Keywords:** cofilin, actin dynamics, post-translational modifications, neuritogenesis, neurodegenerative diseases, cofilin-actin rods

## Abstract

Proteins of the actin depolymerizing factor (ADF)/cofilin family are ubiquitous among eukaryotes and are essential regulators of actin dynamics and function. Mammalian neurons express cofilin-1 as the major isoform, but ADF and cofilin-2 are also expressed. All isoforms bind preferentially and cooperatively along ADP-subunits in F-actin, affecting the filament helical rotation, and when either alone or when enhanced by other proteins, promotes filament severing and subunit turnover. Although self-regulating cofilin-mediated actin dynamics can drive motility without post-translational regulation, cells utilize many mechanisms to locally control cofilin, including cooperation/competition with other proteins. Newly identified post-translational modifications function with or are independent from the well-established phosphorylation of serine 3 and provide unexplored avenues for isoform specific regulation. Cofilin modulates actin transport and function in the nucleus as well as actin organization associated with mitochondrial fission and mitophagy. Under neuronal stress conditions, cofilin-saturated F-actin fragments can undergo oxidative cross-linking and bundle together to form cofilin-actin rods. Rods form in abundance within neurons around brain ischemic lesions and can be rapidly induced in neurites of most hippocampal and cortical neurons through energy depletion or glutamate-induced excitotoxicity. In ~20% of rodent hippocampal neurons, rods form more slowly in a receptor-mediated process triggered by factors intimately connected to disease-related dementias, e.g., amyloid-β in Alzheimer’s disease. This rod-inducing pathway requires a cellular prion protein, NADPH oxidase, and G-protein coupled receptors, e.g., CXCR4 and CCR5. Here, we will review many aspects of cofilin regulation and its contribution to synaptic loss and pathology of neurodegenerative diseases.

## 1. Introduction

The importance of the ADF/cofilin family of proteins with respect to the regulation of actin dynamics in virtually every eukaryotic cell in every kingdom and phyla has resulted in numerous reviews covering most aspects of these proteins, especially within the nervous system where they have essential functions in synaptic plasticity associated with memory and learning [1,2,3]. With so much known about these proteins, why is another review useful at this time? Within the past few years, new post-translational modifications of these proteins have been reported, some of which are isoform specific and, thus, potentially provide independent regulatory pathways. Furthermore, a greater understanding of the mechanisms by which different actin binding proteins modulate ADF/cofilin recruitment and function allows us to build a more integrated picture of their cooperation; thus, re-examining some previously published work and pointing out remaining unanswered questions are also the aims of this review. Starting with the basics of actin dynamics from a structural perspective and building upon this from recent studies that help understand self-regulatory systems, we try to provide context to intracellular studies of actin dynamics, especially as they relate to the formation of cofilin-actin rods. Examining both cytoplasmic and nuclear roles of these proteins is important for further understanding the implications of their sequestration into rods during stress, including either acute stress, such as in ischemic injury, or chronic stress, such as during a progressive degenerative disease. The impacts of cofilin on mitochondrial function also cannot be ignored in neurodegenerative cascades ending in cell death. We end this review with a focus on the signaling requirements for the formation of cofilin-actin rods, how metabolic changes and cooperating proteins help mediate the sequestration of cofilin during rod formation, and whether reversing rods could be beneficial to disease outcomes.

## 2. Actin Dynamics and ADF/Cofilin Basics

**Figure 1:**
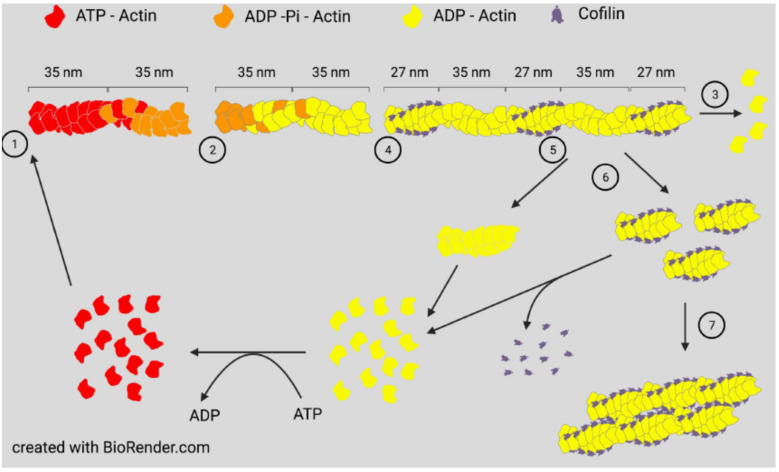
Dynamics of actin assembly and cofilin-mediated disassembly for a filament at steady state. At physiological ionic strength, Mg-ATP-actin subunits add onto the barbed-end of an actin filament **①** and following rapid ATP hydrolysis and slow Pi release **②**; ADP-actin subunits dissociate from the pointed end of a filament to reach a steady state **③**. Filaments not only have a normal crossover point of the two-strand parallel twisted filament of 35 nm but also have a minor stable state with a 27 nm crossover [6,7]. Binding of cofilin to F-actin may initially be attracted to the more highly rotated subunit domain **④**, which is a minor component (~10%) in naked F-actin, or binding may induce a rotation of subunits to this stable state. The “twisted” filament perpetuates the rotation as ADF/cofilin binds along the filament in a cooperative manner [6]. Although binding of a single ADF/cofilin can enhance filament severing in vitro and may do so in vivo if concentrations are low [8], the concentration of ADF/cofilin in mammalian cells is approximately 15–20% of actin [9], and since their actin binding is cooperative [6,10,11], regions of F-actin become saturated **⑤** with severing **⑥** occurring at junctions between saturated and unbound F-actin [12]. F-actin stabilizing compounds (e.g., phalloidin) often used in a fluorescent form to visualize F-actin compete with cofilin for binding F-actin, and they can displace cofilin from actin by stabilizing the untwisted state. Cofilin also competes for F-actin binding with LifeAct, a genetically encoded fluorescent reporter often used for live cell imaging of F-actin [13]. Quantifying intracellular F-actin in fixed cells by measuring bound fluorescent phalloidin will not detect cofilin-actin filaments, which can be quite abundant, as their incorporation into cofilin-actin rods **⑦** exemplifies. In unreviewed work available online at bioRxiv (doi: 10.1101/2021.09.17.460569), Hylton et al. have used cryo-electron tomography to show that neuronal growth cone filopodia contain twisted cofilin-saturated F-actin (27.95 nm crossover) in their proximal region, and they transition into fascin-linked filaments in their distal domain. Packing of cofilin-actin filaments is tighter, leaving no room for fascin to cross-link filaments in this region.

Actin assembly will occur spontaneously in vitro when monomers are above a critical concentration and ionic conditions allow them to self-associate to form a nucleating trimer. Assembly can occur with either ATP-actin or ADP-actin. Two parallel strands of subunits with the same polarity assemble in a helical structure to produce a filament (F-actin). If ADP-actin is assembled, the filament formed is an equilibrium polymer with the same critical (equilibrium) concentration at each end. Filament ends are denoted as barbed or pointed, nomenclature taken from the arrowhead decoration of F-actin by proteolytic fragments of myosin. If assembly is initiated with ATP-actin, the hydrolysis of ATP to ADP-Pi is rapid (~2 s) with a much slower loss of inorganic phosphate (minutes when measured in vitro), the latter being accompanied by a change in the filament structure resulting in different on/off rate constants (and, thus, different equilibrium/critical concentrations) at the slower growing ADP-actin pointed end [4]. Many F-actin binding proteins, including ADF/cofilin, can influence the dissociation rate of Pi [5]. The subunits of ATP-actin continue to add rapidly to the barbed end, but the loss of subunits from the pointed end occurs as the actin monomer pool declines. At a steady state, ATP-actin subunits continue to add to the barbed end, whereas ADP-actin subunits are lost from the pointed end to maintain a constant ratio of filament mass to monomers, but the subunits will continue to treadmill through a filament if ATP is available (Figure 1). In vivo, monomer sequestering proteins prevent spontaneous filament nucleation, so growth occurs from either severed existing filaments or from specific nucleation factors, such as formins or the Arp2/3 complex discussed below.

ADF/cofilin family proteins are expressed in all eukaryotes as essential regulators of actin dynamics [1,14,15,16,17,18]. Mammals express three proteins composed entirely of a single globular ADF homology (ADF-H) domain: ADF (aka destrin), cofilin-1 (aka n-cofilin), and cofilin-2 (aka muscle cofilin), each a separate gene product but sharing >70% sequence identity within an organism. The ADF-H domain has been utilized to make an expanded family of related proteins through gene replication [19]. The domain has ancient roots in archaea [20] and appears to have undergone divergent evolution with ADF/cofilin maintaining greater specificity for actin while a structurally related protein (GMF) coevolved with two actin-related proteins (Arp 2 and Arp 3) for disassembly of the F-actin-nucleating and branching Arp2/3 complex [21,22,23].

All three proteins, ADF, cofilin-1, and cofilin-2, are expressed in adult mammalian neurons [24] and bind both globular (G)-actin and filamentous (F)-actin with a strong preference (>40 fold) for binding ADP-actin [25,26]. At low concentrations with respect to actin subunits (1:750), cofilin is an effective F-actin severing protein. At higher concentrations, severing efficiency declines but occurs at the junction between cofilin-saturated and unsaturated regions, which are sites of increased strain [12,27], but stable cofilin-actin complexes can also nucleate growth [9,26] (Figure 1).

There are subtle differences in how mammalian ADF and cofilin-1 modulate actin, and although each can rescue many functions of the other in cell behavioral assays, there are some isoform specific functions [28]; these could arise because of differences in the protein’s inherent activity or reflect differences in their sites of translation or cellular regulation. Inherent activity differences are observed in the assembly of ADF-actin and cofilin-actin complexes, each of which assembles into filaments that appear identical to F-actin saturated with cofilin or ADF, but the critical concentration for assembly of ADF-actin is >5 μM, whereas it is ~1 μM for cofilin-actin, which is approximately the same as for ADP-actin alone. Thus, ADF-actin may contribute to the intracellular pool of monomer, whereas cofilin-actin will not [11,26,29]. For similar reasons, cofilin-actin filaments might survive longer in cells where stress has resulted in a reduction in ATP and filaments approach their equilibrium state.

Genetic knock out (KO) of cofilin-1 in mice is embryonic lethal [30], whereas knock out of ADF has minimal observable effects on overall development. ADF KO mice do have one striking defect in being unable to downregulate corneal epithelial proliferation, resulting in corneal thickening and blindness and giving rise to the name associated with this mouse line, Corn^1^ [31,32,33]. The expression of cofilin in corneal epithelium is insufficient for rescue of deficits. However, deficits are largely rescued by genetic inactivation of the Serum Response (transcription) Factor (SRF), implying a significant role for aberrant gene expression in response to loss specifically of ADF in this tissue [34].

The question of how expression of ADF and cofilin is controlled is also of great interest. During myoblast differentiation to myocytes in chickens, which only express ADF and cofilin-2, there is a big switch in expression to cofilin-2 as the myocytes mature [35]. There is also a big change in the expression of the skeletal muscle α-actin isoform and in the assembled actin pool, raising the question “is ADF or cofilin expression tied to the monomeric actin pool?” This was addressed in mouse C2C12 myoblasts by expressing a mutant form of actin that could not assemble into filaments. We previously proposed that ADF expression but not that of cofilin is downregulated by a post-transcriptional mechanism and the expression of total actin is also controlled [36]. This feedback control is modulated by the actin monomer pool as depolymerizing actin filaments using latrunculin A also decreases ADF but not cofilin expression. ADF, cofilin, and various actin isoforms are known transcriptional targets of the SRF and its cofactor MKL1 [37]. The activation of MKL1/SRF gene transcription is closely coupled to free cellular G-actin levels [38], suggesting an alternate mechanism for linkage between ADF expression and the actin monomer pool. Differential regulation of individual SRF target genes through miRNAs or competing repressive factors may contribute to distinctive ADF vs. cofilin expression mediated by SRF [39].

The ubiquitous cofilin-1 isoform accounts for 80–90% of the total ADF/cofilin in rodent neurons [40,41] and human brain [42], with the highest levels expressed in neurons and microglia. Hereafter, we will just refer to cofilin unless a distinction between isoforms is required. Of note, chickens do not express cofilin-1, and ADF is the ubiquitous isoform in chicken neurons [43].

## 3. Dynamic Regulation of Actin Assembly by Cofilin

### 3.1. Self-Regulation

Before discussing the many post-translational modifications of cofilin that can regulate its actin binding, it is worthwhile to understand how cofilin-mediated debranching, severing, and depolymerization of F-actin networks can be self-regulated. The first such in vitro system utilized the comet tail motility of *Listeria monocytogenes* [44], which was found to function in a cytoplasmic protein mixture from *Xenopus* oocytes by using fixed (dead) *L. monocytogenes* [45]. Motility required the activation of the Arp2/3 complex by a bacterial protein ActA [46], which induces a conformational change bringing together the two actin-related proteins Arp2 and Arp3 to form a structure that appears, from the perspective of a free actin monomer, to be a filament barbed end and elongation then ensues. The activated Arp2/3 complex, which remains at the pointed end of the actin, can bind along the side of filaments to generate a 70° branched network, forming a comet shaped tail with subunit addition occurring near the bacterium as branched filaments are capped during their short period of growth. Maintenance of the short comet tail depends on the presence of cofilin in the ooplasm to disassemble the actin array from its distal end [47]. A fully reconstituted in vitro system using purified proteins showed an absolute requirement for actin, Arp2/3 complex, cofilin, and capping protein, but a greater effective movement was obtained if profilin (an actin monomer binding protein), α-actinin (F-actin cross-linking protein), and vasodilator stimulated phosphoprotein (VASP), an enhancer of barbed-end actin polymerization that delays filament capping [48], were also added [49]. Other pathogens also utilize an array of actin assembly mechanisms to hijack the invaded cell’s machinery for their own locomotion, including ones that assemble linear (unbranched) filaments [50].

**Figure 2:**
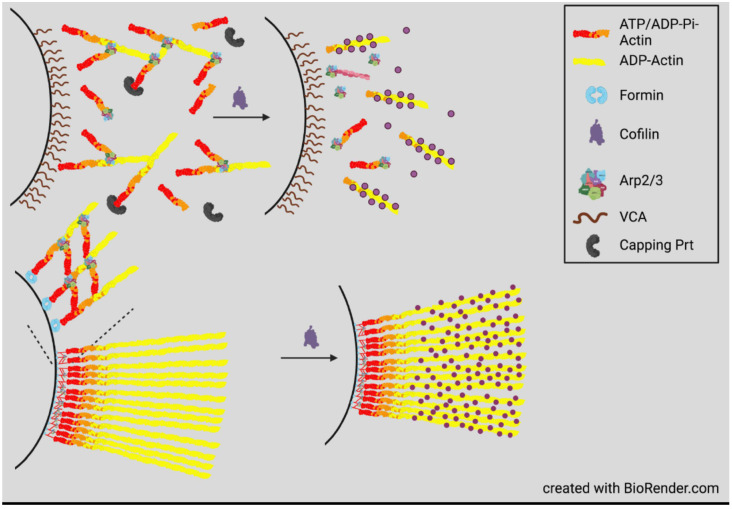
Effects of cofilin on different actin filament systems in vitro. (**Top**) Diagrammatic representation of an Arp2/3 nucleated branched actin filament network at the surface of a bead with an attached Arp2/3 activator showing activation of Arp2/3 complex and nucleation of filaments, which bind through the complex to the side of older filaments to generate a branched network. Filaments are disassembled by cofilin as they age (loss of Pi) relative to ADP-actin and show array treadmilling. Capping protein limits growth to short filaments; profilin (not shown) sequesters monomers to prevent spontaneous nucleation; CAP1 (not shown) enhances turnover of cofilin-actin fragments. Under low ionic strength conditions, the branched network is rapidly disassembled even by low cofilin concentrations and will not assemble above 50 nM cofilin [51]. (**Bottom**) Cofilin can maintain a steady state dynamic actin network mediated from both Arp2/3 complex and formin-nucleated filaments in the absence of cofilin post-translational regulation. Beads maintain a narrow band of Arp2/3 complex branched filaments along their surface with long, linear formin-nucleated filaments extending in a halo and binding excess cofilin. Beads start rotating, probably because of asymmetry in assembly, and maintain rotation. Based on data from Bleicher et al. [51].

One question remaining from these studies is whether there is any difference in how cofilin turns over branched and linear filament systems when they occur together? This question was addressed by examining how cofilin regulates the assembly dynamics of actin networks around beads where their surface is modified with various actin assembly nucleators, including either or both an Arp2/3 activator and a formin [51] (Figure 2). Experiments were performed in the presence of F-actin barbed-end capping protein to limit the elongation of Arp2/3 branched filaments; profilin-1 to suppress spontaneous nucleation of filaments and to provide the profilin–actin complex with the ability to increase the efficiency of formin-nucleated filament growth [52]; and cyclase-activated protein-1 (CAP1) to aid in the turnover of cofilin-severed filaments and to promote the exchange of ATP for ADP on the released actin monomers [53,54,55]. Beads containing only formin nucleated a broad halo of filaments that was decorated with cofilin, except in the region near the bead where ATP and ADP-Pi subunits dominated.

Two caveats in applying these results to intracellular behaviors are as follows: (1) the studies were performed well below physiological ionic strength to allow for slower and measurable changes in actin assembly dynamics; and (2) formin-nucleated filaments that have their free rotation hindered by cross-linking generate a rotation or torsional twist of the filament that is opposite to that induced by cofilin binding and, thus, are resistant to cofilin [56]. Thus, although these results suggest that cofilin and its associated proteins developed as a self-regulated system for filament turnover requiring none of the post-translational modifications that regulate intracellular control in metazoans, evidence is lacking for showing that this self-regulating system works under intracellular conditions. However, many simple single cell eukaryotes encode for only one member of the ADF/cofilin family and in many cases may function without the need for post-translational modification. The sequestering of excess active cofilin along linear filaments suggests an alternative method for maintaining optimal cofilin levels depending upon the amounts of actin nucleators, the local distribution of linear and branched filaments, and the presence of other co-factors that modulate cofilin severing and depolymerizing activities, rather than a complete reliance on post-translational regulation. Alternatively, there is also an array of F-actin binding proteins discussed later that can protect filaments from the action of cofilin.

### 3.2. Direct Regulation by Lipid Binding

**Figure 3:**
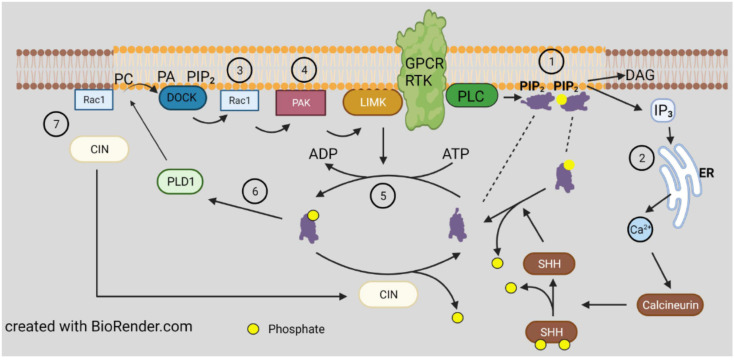
Membrane organization of cofilin regulatory proteins and lipid binding sites. Membranes associated phosphatidylinositol phosphates (PIPs), found in highest concentrations in sphingolipid/cholesterol enriched lipid raft domains, bind both cofilin and serine 3 phospho-cofilin **①**, which are released upon activation of PLC. Different isoforms of phospholipase C (PLC) can be activated by receptor tyrosine kinases (RTKs, diagrammed here) or by G-protein coupled receptors (GPCRs) and release diacylglycerol (DAG) and various phosphoinositols. **②** Inositol 1,4,5 triphosphate (IP_3_), released from PI(4,5)P_2_, (PIP_2_) is an activator for release of intracellular Ca^2+^. Both GPCRs and RTKs also activate pathways for stimulating guanine nucleotide exchange factors (GEFS) for activating Rho family GTPases (e.g., Rac1) **③**, which work at the plasma membrane to activate PAKs **④** by binding to their autoinhibitory domain to permit autophosphorylation that activates the PAK. Active PAK phosphorylates and activates LIMK1, also membrane bound, to locally inactivate cofilin **⑤**. Cofilin phosphorylated on S3 is an activator of phospholipase D1 **⑥**, which converts phosphatidylcholine, a major membrane phospholipid, to choline and phosphatidic acid (PA) [61]. PA can further signal through binding to other membrane proteins, such as those with DOCK domains, to enhance or reduce downstream signaling. The cofilin phosphatase chronophin (CIN) is recruited to the leading edge of cells through a Rac1 **⑦** and PI3-kinase dependent pathway [62].

The first and simplest mechanism discovered for regulating cofilin activity is its inhibition by binding to phosphatidylinositol phosphates (PI-4P and PI-4,5P_2_; PIPs) [57], which are important lipid signaling molecules within the cytoplasmic leaflet of vesicles and plasma membrane (Figure 3). Although not widely embraced at that time as a regulatory mechanism, the discovery of two cofilin-dependent phases of increased F-actin barbed-end transients in epidermal growth factor (EGF) stimulated breast cancer cells changed this viewpoint [58]. The peak of F-actin barbed-end formation occurring 1 min after EGF treatment requires phospholipase C (PLC) to release cofilin from the membrane [59], whereas a second transient peak occurring 3 min after EGF treatment is dependent on cofilin dephosphorylation, discussed further below. Since the many isoforms of PIPs sort to different endosomal and vesicular membrane compartments, cofilin binding or release accompanying modifications in the PIP composition of the cytoplasmic leaflet could restructure the local actin filament network around vesicles for their transport to or fusion with other compartments [60].

### 3.3. Post-Translational Regulation of Cofilin

**Figure 4:**
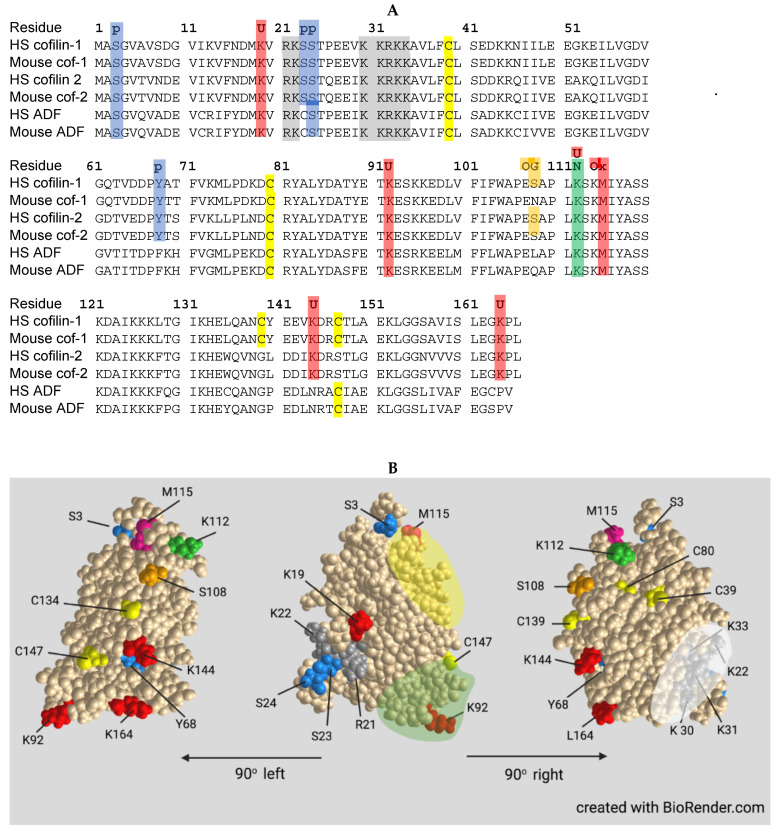
Primary sequence comparison and 3D structure of ADF/cofilins. (**A**): Aligned sequences of human (HS) and mouse cofilin 1, cofilin 2, and ADF, identifying sites for regulation and modification discussed in the text: (p-blue) phosphorylation; (U-red) ubiquitinylation; (gray highlight) bipartite nuclear localization sequence; (OG- orange) O-GlcNAcylation; (N-green) neddylation (also a ubiquitinylation site); and (Ox) Methionine oxidation. Cysteines are highlighted in yellow: C39-C147 forms an intermolecular disulfide found in rods, and C39-C80 and C139-C147 form intramolecular disulfides in response to oxidative stress that targets cofilin to mitochondria [63,64,65]. Residues that could account for differences in regulation between isoforms or between human and mouse are 68, 108, 139, 144 and 164. (**B**): Structure of human cofilin-1 viewed facing the actin-binding side with 90° rotations for side views. Residue colors match as close as possible with those used in A. Green highlight in center image is the lower F-actin interface, and yellow highlight is the upper F-actin–G-actin interface. Sequence data and 3D protein structure from NCBI using Cn3D software version 4.3.1.

Unless otherwise noted, the post-translational regulatory mechanisms described below apply to all three isoforms. The sequences around post-translational regulatory sites are highly conserved, with exceptions being noted (Figure 4). It should also be noted that major discoveries over the past decade have shown that several other actin binding proteins dramatically influence the ability of ADF/cofilin to bind, sever, and turnover subunits of F-actin, but preferences for specific ADF/cofilin isoforms have not been determined in many instances.

### 3.4. Phosphorylation

Evidence for phospho-regulation of ADF/cofilin family proteins first came from studies of extracts from chicken myocyte cultures that showed two species of ADF on both 1D and 2D immunoblots, one of slightly higher mass and more acidic which disappeared after phosphatase treatment [35]. Only the species insensitive to phosphatase bound to actin, and it accounted for between 15 and 60% of the total ADF from a variety of tissues of chick and rodent cells lines. The location of the single phosphorylation site was identified as Ser3 on the encoded protein (Ser2 on the demethionated and N-Acetylated protein) for both ADF and cofilin [66,67] (Figure 4).

The first kinase identified to phosphorylate cofilin S3 is the ubiquitous LIM kinase (LIMK) [68,69] for which two isoforms exist. A second kinase family with a structurally related kinase motif was identified in testes (TESK1 and 2) [70] and a germinal center kinase, NRK/NESK, active in embryogenesis was also shown to enhance cofilin S3 phosphorylation [71]. LIMK1 is palmitoylated on its di-cysteine motif (C7 and C8), absent in LIMK2, resulting in LIMK1 membrane anchoring, which is required for efficient phospho-activation at its T508 by members of the p21-activated kinase (PAK) family [72] (Figure 3). These, in turn, are activated by binding to the isoprenylated membrane anchored GTPases, Rac, Cdc42, and others, which are also, in turn, activated by GTP exchange factors (GEFs), many of which are either transmembrane receptors or proteins recruited to the cytoplasmic side of transmembrane receptors [73]. The binding of the GTP forms of Cdc42 or Rac to PAKs relieves their autoinhibition and allows their self-activation by phosphorylation of a specific serine (e.g., S474 in PAK4). LIMK2, which is not membrane bound, is activated by phosphorylation on T505, equivalent to T508 in LIMK1.

Rho kinase (ROCK) is the effector of the activated GTPase Rho A. ROCK can also phosphorylate and activate LIMKs. Effectors of the different Rho family GTPases often have opposing effects on downstream pathways. For example, ephrin A working through its Eph receptor and its nucleotide exchange factor ephexin activates RhoA and ROCK but inhibits the Cdc42/Rac1-PAK signaling pathway [74]. These opposing activities can help explain some otherwise confusing results. PAK4 is autophosphorylated on S474 (activation) and then phosphorylates its effectors, which includes LIMKs [75]. However, knockout of Cdc42 in neurons results in the expected decline in PAK phosphorylation but results in an increase in phosphorylated and active LIMK as well as in phosphorylated cofilin [41]. Since, as we will see further along, the formation of cofilin actin rods in neurons requires active (dephosphorylated) cofilin, it is not surprising to find a significantly reduced rod response in neurons expressing dominant negative (N17) Cdc42 and a rod increase in neurons expressing constitutively active (V12) Cdc42 [76].

An additional serine phosphorylation site in cofilin that inhibits its activity was identified as S23 and/or S24 catalyzed by protein kinase Cα (PKCα) [77]. This modification brings about an increase in F-actin that results in the cessation of histamine release following stimulated degranulation of basophilic leukemia cells. The expression of the non-phosphorylatable S23,24A mutant, which binds, severs, and depolymerizes F-actin, increases degranulation, whereas expressing the phosphomimetic S23,24E form does not. Global screening of all cofilin phosphorylation sites in myeloid cells not only identified S3 as the major phosphorylation site but also identified minor sites of phosphorylation as S24, S41, S108, and S156 as well as on tyrosines Y68, Y82, and Y140 [78]. Y68 phosphorylation targets cofilin for ubiquitinylation and degradation (discussed below). Little is known about the contribution, if any, of phosphorylation at S41, S156, Y82, or Y140 relative to cofilin activity or cellular function. Phosphorylation at these other sites is insensitive to inhibitors of LIMK. Direct phosphorylation of cofilin-1 on T25 by extracellular signal-regulated kinase (ERK1/2) has been shown to alter cardiac actin dynamics in dilated cardiomyopathy [79]. Increased cofilin-2 phosphorylation on S23/24 has also been reported to lead to myocardial aggregates in dilated cardiomyopathy [80].

**Figure 5:**
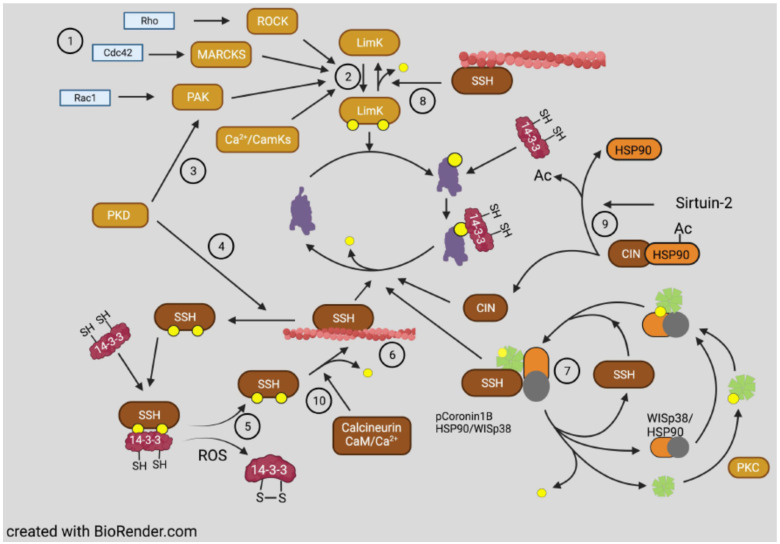
Major pathways for phospho-regulation of cofilin. **①** Upstream activation of PAKs by Cdc42/Rac relieves autoinhibition allowing self-phosphorylation of ser474 (PAK4) with subsequent phosphorylation (activation) of LIMK1/2 (T508 and T505, respectively) **②**. Protein kinase D1 (PKD) **③** phosphorylates PAK4 on ser99, which mediates its binding to 14-3-3 (not shown) and recruits it to sites of LIMK1 at the membrane [90,91]. Two sites in the C-terminal tail domain of SSH1L are phosphorylated. PKD phosphorylates SSH1L on S978 **④**, which enhances binding of 14-3-3 to inhibit dephosphorylation by non-specific phosphatases and inhibits its F-actin binding through its tail domain, one mechanism by which its N-terminal cofilin phosphatase activity is activated [92,93]. PKD isoforms, thus, serve as rapid inactivators (phosphorylation) of localized cofilin activity [91,94]. Removal of 14-3-3 from phospho-SSH1 **⑤** can be achieved by oxidation (peroxide or ROS from NOX), causing disulfide bond formation within 14-3-3 [95]. SSH1 requires binding to F-actin **⑥** or **⑦** to a complex of phospho-coronin1B (green molecule) bound to WISp38/Hsp90 [89]. Active SSH1 also serves to dephosphorylate LIMK1 on T508 **⑧**, which can bring about a rapid increase in active cofilin [88]. Much of CIN in neurons is held in an inactive form by hsp90 but can be released in an active state **⑨** by hsp90 inhibitor 17-AAG [96] or possibly by sirtuin-2 (an HDAC) deacetylation of hsp90 [97]. Calcineurin, a Ca^2+^/calmodulin-activated phosphatase, also activates **⑩** SSH1 [98], the likely mechanism of SSH1 activation through integrin/RanBP9 [99].

Cofilin dephosphorylation (activation) in vitro can be catalyzed by some non-specific phosphoprotein phosphatases, but in vivo phosphorylated cofilin is complexed with isoforms of 14-3-3 (mainly ζ), which generally restricts phosphate accessibility to these ubiquitous but non-specific protein phosphatases [81]. Localized dephosphorylation in cells is mainly catalyzed by two unrelated phosphatases: those of the slingshot (SSH) family [82] and pyridoxal-5-phosphate phosphatase, also named chronophin (CIN) [83]. A limited number of phospho-protein substrates have been identified for CIN, consisting of cofilin [83], phosphorylated steroid receptor coactivators 1 and 3 [84], and neurofibromin 2 (aka merlin), a tumor suppressor protein encoded by the NF2 gene mutated in neurofibromatosis type 2 [85]. SSH has three major members, two with long and short isoforms of which SSH1L is the most ubiquitously expressed [82,86]. SSH also has a limited group of substrates but in addition to pS3 of cofilin, it includes pS2 of coronin 1B [87], further discussed later, and pT508 of LIMK1 [88] generating a cofilin activation cascade (Figure 5). The abundant SSH1L requires binding to F-actin for activity, or it can be activated when bound to a complex of phospho-coronin1B [89], shown in Figure 5 but discussed further below.

Chronophin, an unusual phosphatase, forms a mixed anhydride intermediate on residue D25. The enzyme functions as a dimer with homophilic interactions required for catalysis [100]. In neurons, CIN is found bound to the chaperone Hsp90 in an ATP-dependent manner [96]. Interestingly, sirtuin2 (SIRT2), a tumor suppressor and member of the class III histone deacetylase (HDAC) family, suppresses actin polymerization in cancer cells through deacetylation of Hsp90, resulting in increased dephosphorylation of cofilin [97], a process consistent with CIN participation (Figure 5).

Locally regulating cofilin activity is essential for the development of cell polarity, maintenance of polarized cell migration, and changing directions of migration in response to extracellular signals, such as during growth cone (GC) guidance (pathfinding). Cells maintain a network of membrane proximal F-actin directly adjacent and parallel to the membrane and by tethering it via transmembrane and cytoplasmic leaflet associated F-actin binding proteins, restricts the formation of filopodial and lamellipodial membrane protrusions necessary for motility [101]. In a normal migrating cell, the amount of membrane-proximal F-actin is higher in the rear than in the front, whereas the total F-actin measured by staining with fluorescent phalloidin has the opposite distribution. Cofilin can break down the membrane proximal F-actin and is regulated in typical cultured mammalian cells undergoing the development of polarity by altered localization of active LIMK1 (pSer508) relative to the rear of the cell and active SSH relative to the front of the cell [99], creating gradients of cofilin activity from high in the front and low in the rear. However, this may not be the only mechanism. Depolymerizing the actin cytoskeleton in endogenously polarized chick fibroblasts with latrunculin A, a monomer sequestering agent, allows one to follow the process of repolarization following washout. Cells form a completely symmetrical lamellipodium with repolarization visualized morphologically starting with lamellipodial collapse at what will become the cell rear and the remaining lamellipodium becoming the leading edge as migration ensues. Myosin II activity is required to create aligned actin filament bundles necessary for repolarization, but it is the ADF-driven actin disassembly that drives the collapse of the cell rear and formation of the leading edge [102]. These events likely occur because of a balance in myosin II and ADF/cofilin competition in binding F-actin [103,104] and perhaps because of the need for SSH1L to bind F-actin for its ability to activate cofilin. Such events result in cytoplasmic regions devoid of F-actin and other regions enriched in contractile filaments, a distribution that becomes immediately obvious in cells silenced for lamin A/C, which organizes nuclear envelope linkages to cytoplasmic stress fibers [105].

Although cofilin phosphorylated on S3 is considered inactive, that is correct only in so far as its ability to bind G- and F-actin. S3 phosphorylated cofilin is an activator of phospholipase D1 (PLD1), an enzyme that hydrolyzes membrane phospholipids to phosphatidic acid [61], which has important roles in membrane signaling impacting an enormous variety of cell behaviors [106]. There are two mammalian isoforms of PLD, with PLD1 generally localizing to intracellular membranes and translocating to the plasma membrane upon activation and PLD2 which localizes to the plasma membrane and is internalized to other domains upon its activation. The specificity of phospho-cofilin for the activation of only PLD1 suggests that the sites of active LIMK1 might also serve to recruit p-cofilin-activated PLD1, which can further signal through its interactions with membrane PI(4,5)P_2_ and Rho family GTPases [106] (Figure 3).

### 3.5. Ubiquitinylation and Neddylation

**Figure 6:**
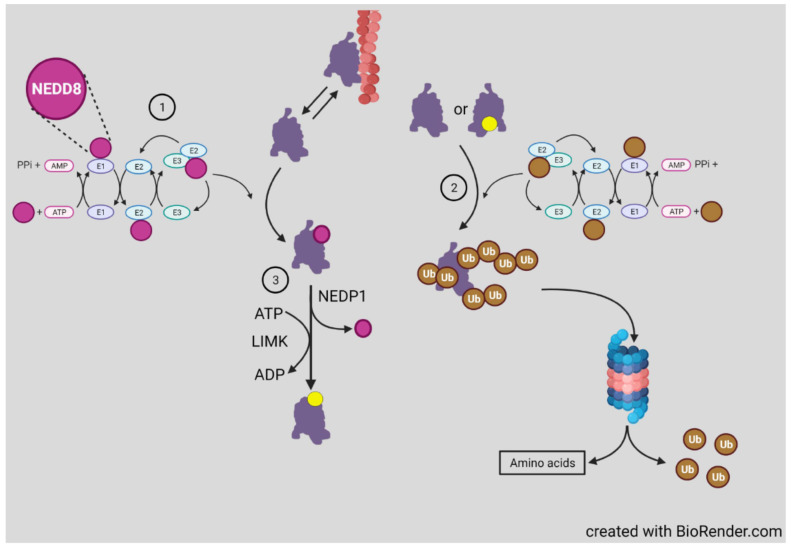
Enzymatic cascades for ubiquitinylation and neddylation of cofilin. **①** Specific enzymes (E1), for which there is often only a single isoform, activate the polypeptide modifier, ubiquitin, or NEDD8 through a thioacyl derivative on its C-terminus linking it to E1. The activated polypeptide is then transferred to an E2 enzyme for which a few different isoforms are expressed. The E3 enzyme, which exists as multiple isoforms with different substrate recognition capabilities, binds both its substrate and the E2-polypeptide and transfers the polypeptide to its lysine acceptor in the substrate. Neddylation targets cofilin on only K112, preventing it from rapidly rebinding F-actin and allowing time for its phosphorylation [108]. **②** Cofilin K112 and lysines 19, 92, 144, and 164 are ubiquitinylated, a process enhanced by phosphorylation of Y68 by vSrc tyrosine kinase, resulting in more rapid cofilin degradation by the proteasome [107]. The NEDD8 activating enzyme is inhibited by MLN4924, and NEDD8 is removed from substrates **③** by the enzyme NEDP1. Inhibition of neddylation results in a large increase in F-actin bound cofilin due to its decreased phosphorylation.

Post-translational modification of proteins on lysine residues with different families of ~8 kDa polypeptides is a recurrent regulatory mechanism in protein turnover and function. Among the most common families of polypeptide modifiers include ubiquitin and NEDD8, each of which utilizes a similar sequence of three enzymes specific for the modified polypeptide (Figure 6). Cofilin is targeted for proteosome degradation by ubiquitinylation, which is enhanced by vSrc phosphorylation of Y68 [107] (a residue that is absent in ADF), suggesting that the turnover of these proteins may be differentially regulated. Cofilin activity could also be regulated by reversible ubiqutinylation at any one of its several known ubiquitinylated lysines [108].

Cofilin is also a target of neddylation, a modification first discovered for E3 ligases associated with cell cycle regulation and, thus, is most extensively studied in cancer [109,110]. In an unbiased assay that allowed proteins (and peptides derived therefrom) modified by either or both NEDD8 and ubiquitin to be isolated, several hundred neddylated and/or ubiquitinated proteins were identified in HEK293 cells and mammalian neurons [108]. Among these were cofilin and ADF and only a single lysine (K112) was neddylated. K112 is highly conserved across mammalian ADFs and cofilins (Figure 4). Other cytoskeletal associated proteins, some of which are known to coregulate actin dynamics with cofilin, were also identified in the unbiased screen, but cofilin was the only one further examined for the role of its neddylation in neurons. K112 is located on the actin binding α-helix 4 where neddylation inhibits cofilin-F-actin binding. Cofilin neddylation is short lived and almost impossible to detect in cell extracts unless the expression of the deneddylating enzyme NEDP1 is silenced [108]. Non-neddylatable cofilin^K112R^ expressed in the absence of endogenous cofilin remained unphosphorylated and associated with F-actin, suggesting that cofilin released during actin turnover requires transient neddylation to delay its rebinding to F-actin allowing for its phosphorylation, which might require diffusion to the plasma membrane where LIMK1 is localized. Actin filament turnover in cells expressing the non-phosphorylatable mutant cofilin^(S3A)^, which retains its actin binding and severing activities, behaves identically to cells expressing cofilin^(K112R)^ in being unable to effectively mediate filament turnover, supporting the hypothesis that phosphocycling of cofilin, rather than the relative amounts of cofilin that are phosphorylated or unphosphorylated, drives actin dynamics. This hypothesis was suggested over 20 years ago from the finding that dephosphorylation rates of ADF and cofilin dramatically increased without any change in the ratio of phospho to dephospho forms of either following the restoration of membrane ruffling in quiescent cells by addition of serum [111].

Global inhibition of neddylation using an inhibitor (MLN4924) of the E1 NEDD8-activating enzyme decreases the ability of hippocampal neurons to undergo the normal transition between stage 1, sprouting of neurites, to stage 3, in which a presumptive axon has been morphologically established. Disrupting actin with cytochalasin D or stabilizing microtubules with taxol increase the proportions of stage 3 neurons [108], but because so many different proteins undergo transient neddylation, a more targeted approach was applied to study cofilin neddylation. The inhibition of the NEDD8 activating enzyme had no effect on cofilin expression levels but it did decrease the amount of phosphorylated (inactive) cofilin, as did the expression of cofilin^(K112R)^ (the non-neddylatable mutant). Surprisingly, cofilin^(K112R)^ and wild type (WT) cofilin in the presence of MLN4924 maintained their interactions with SSH1L, LIMK1, and CIN, as measured by co-precipitation with biotin-tagged cofilins. As mentioned previously, cofilin binds cooperatively to F-actin with severing occurring at the junctions between cofilin saturated and unbound regions [12,112]. In the presence of the neddylation inhibitor, F-actin binding of both cofilin^(K112R)^ and WT cofilin increased. In cells silenced for ADF and WT cofilin, the ratio of pelleted actin to total actin in lysates increased significantly, as expected. Re-expression of WT cofilin reduced pelleted actin levels toward those in cells with only ADF knocked down. Surprisingly, the expression of cofilin^(K112R)^ in the ADF/cofilin-knock down cells reduced pelleted actin nearly identically to cells in which cofilin^(S3A)^ is re-expressed [108]. Taken together, these findings suggest that the absence of phospho-regulation is occurring in both cofilin^(K112R)^ and cofilin^(S3A)^ expressing cells and that non-phosphorylatable forms of cofilin are more stably associated with the pelletable (sedimented at 100,000 g for 30 min) actin pool. An even greater binding to F-actin might have been found if sedimentation conditions were more stringent since smaller pieces of F-actin decorated with cofilin, such as those produced in F-actin depolymerization assays, will not be fully cleared by the typical centrifugation conditions used in pelleting assays [26]. Thus, stable small fragments of cofilin-saturated F-actin likely contribute significantly to the content of the soluble actin pool in cells expressing either cofilin^(S3A)^ or cofilin^(K112R)^ [6,8,26].

The functional importance of cofilin neddylation to neuronal development was demonstrated in dissociated neurons in which expression of endogenous ADF and cofilin was silenced on the day of plating [108]. Typically, cofilin and ADF have half-lives in cells of about 24 h, with a reduction to about 10% of control levels requiring 72 h [28,41]. Thus, a decline in neurite length is slow to develop and averaged only about 40% by 4–6 days in vitro (DIV). Outgrowth was rescued by including a vector for WT cofilin during nucleofection but was only slightly improved by expressing the cofilin^(K112R)^. Similar studies were performed in developing mouse brain by in utero electroporation with the same plasmids used in neuronal culture. Upper layer cortical neurons showed reduced apical dendrite length, rescued by the inclusion of cofilin WT but not by the inactive phosphomimetic cofilin^(S3E)^ or cofilin^(K112R)^ vectors [108]. These results again suggest that inhibition of cofilin phospho-regulation impairs neuritogenesis. It would be of interest to study this rescue by expressing WT cofilin containing mutations that selectively disrupt either its severing (S94D) or depolymerizing (Y82F) activities to determine which activity, if either, is most required.

Neddylation also occurs in dendritic spines, the postsynaptic densities of excitatory neurons in mammalian brain, and is required for spine development and maturation [113], including clustering of metabotropic glutamate receptor mGlu7 [114]. Although many spine proteins are neddylated, the importance of cofilin to spine plasticity in learning and memory [3,115,116,117] make it a likely contributing effector of the neddylation response in spines and well worth studying with cofilin^(K112R)^.

### 3.6. Oxidation/Reduction (Redox) Regulation

Cofilin has four cysteine residues and maintains its actin dynamizing activity and monomeric form if sufficient reducing reagents are present [118]. However, under increased oxidative stress, cofilin dimerizes both in vitro [118] and in vivo [65,119] through the formation of an intermolecular disulfide bond between C39 and C147 (Figure 4). The formation of dimers is favored for cofilin bound to monomeric ADP-actin [119]. Disulfide bonds within cofilin (intramolecular) between C39/C80 and C139/C147 form in vivo under oxidizing conditions induced by peroxide or the naturally produced oxidizing agent taurine chloramine and alter cofilin’s cellular localization, particularly its accumulation at mitochondria [63,64]. Neither cofilin-2 nor ADF has a cysteine at 139 and, thus, will not form the second internal disulfide (Figure 4). Oxidation induces loss of cofilin’s actin-binding activity, but this is not due to cysteine oxidation but rather by oxidation of methionine 115 to sulfoxide, a reaction that is reversable by methionine sulfoxide reductases [120]. Indeed, many thiol oxidizing agents, including the large family of cucurbitacins, induce alterations in the actin cytoskeleton through cofilin dimerization/oxidation [121,122].

Both actin and cofilin have cysteine SH groups that are substrates for direct S-glutathionylation, a major defense mechanism against oxidative damage in neurons. Actin is modified on its penultimate residue, C374, whereas each of cofilin’s 4 cysteine residues can be modified by glutathione [123,124]. S-Glutathionylation of cofilin reduces its ability to depolymerize F-actin, and S-glutathionylation of actin reduces its rate of polymerization, but both proteins recovered full activity after dethionylation. Significant changes in the levels of cofilin S-glutathionylation have been found in regions of rat brain that respond by dendritic spine enlargement to cocaine-conditioned environmental cues, suggesting a physiological role for this cofilin modification in synaptic function [124].

Proteins that regulate cofilin are also susceptible to ROS-dependent changes. One such protein is PKD1, the activator of PAK4 and inhibitor of SSH1L shown in Figure 5. PKD1 is a ROS sensor that is activated in response to elevated ROS and conveys the information to the nucleus for upregulation of antioxidant genes [125]. Many targets of PKD1 could potentially mediate the nuclear response, among them being active cofilin, through its nuclear transport of actin, discussed later.

### 3.7. O-GlucNAcylation

Protein modification by ser/thr-linked N-acetylglucosamine (O-GlcNacylation) [126] is catalyzed by O-GlcNAc transferase (Ogt) and its removal by O-GlcNAcase (Oga) [127]. O-GlcNAcylation is a highly conserved post-translational process occurring across phylogeny, and more than 5000 human proteins undergoing O-GlcNAcylation have been identified [128]. Ogt is necessary for embryonic development, stem cell viability [129], and corticogenesis in the brain [130], but it controls the pool of neural/stem/progenitor cells through a Notch-signaling pathway even into adulthood [131]. Ogt levels in mouse brain decline during aging but increasing its expression improved cognitive function in aged mice [132]. The role of specific O-GlcNAcylated proteins in mediating Ogt regulated processes is very much understudied.

O-GlcNAc-cofilin was found in rat brain extracts by immunopurification with an anti-*O*-GlcNAc antibody [133]. The identification of Ser108 as the substrate site for Ogt was determined in human cancer cells by site specific mutagenesis [134]. In both human and rat breast cancer cell lines, the cofilin O-GlcNAcylation facilitates its localization to invadopodia, which are sites of matrix metalloprotease release and actin-dependent protrusions associated with cancer metastasis [135]. Cofilin ser3 phosphorylation and O-GlcNAcylation are independent of each other [134]. The expression of the S108A mutant (non-*O*-GlcNAcylated) does not alter F/G-actin ratios supporting their independent roles. Although migrations of breast cancer cells expressing the S108A mutation are normal in a 2D migration assay, they show diminished ability to cross a matrigel layer in a 3D transwell migration assay commonly used to measure invasive potential, suggesting O-GlcNAcylated cofilin has some specialized function in invadopodium formation or delivery/secretion of matrix metalloproteases. Neuronal growth cones also secrete metalloproteases [136], so it will be of interest to determine if cofilin O-GlcNAcylation has a function in growth cone pathfinding in vivo. Neither mouse cofilin-1 nor mammalian ADFs have a modifiable serine or threonine at this position (or within ±4 residues; Figure 4), making it unlikely that they are directly modified by Ogt. The absence of S108 in ADF might explain subtle differences observed between the ability of ADF and cofilin-1 to support different aspects of membrane protrusion events followed in a rat breast tumor cell line [28]. Perhaps more importantly, it calls into question interpretations made from mouse models studying tumor metastasis in which cofilin regulation via O-GlcNAcylation does not occur and, thus, may differ from rats and humans in pathways regulating invadopodium formation/function.

Since neither mouse ADF nor cofilin-1 has a serine at residue 108, studies of the importance of cofilin-2 O-GlcNAcylation in neuronal growth and pathfinding can be readily investigated in mouse neurons, and there are several studies suggesting it could be of interest. For example, increased O-GlcNAcylation targeted to dopaminergic neurons prevented their degeneration in a mouse model of Parkinson’s disease (PD) [137] and ameliorated the pathological degeneration of neurons in Alzheimer’s disease (AD) mice [138]. Increasing O-GlcNAcylation in both young and old mice was neuroprotective with respect to ischemic stroke [139]. Additionally, a decline in O-GlcNAcylated proteins occurs in spinal cords of mouse models of Amyotrophic Lateral Sclerosis (ALS) during motor neuron atrophy and in mice deficient in a reactive oxygen species (ROS) sensor (NPGPx), a direct modulator of O-GlcNAcase [140]. The vast array of Ogt substrates that relates to the areas discussed below includes transcription factors [141], the microtubule binding protein tau [142], amyloid precursor protein (APP) [143], and Drp1 [138]. How Ogt modification of these substrates affects the associated disease process has yet to be established. Since O-GlcNAc of cofilin-2 could allow its independent regulation from ADF and cofilin-1 (Figure 4), understanding its role in many of the disease models could prove fruitful.

## 4. Other Proteins Modulating Cofilin Activity and Their Regulation

We have already discussed the roles of several proteins that work in conjunction with cofilin to regulate the organization and turnover of F-actin when describing a cofilin-actin self-regulating system above. Among these are the ubiquitous F-actin capping protein and the monomer binding protein profilin-1 (Figure 2). Capping protein can not only be sequestered by cytoplasmic binding to myotrophin [144] but also has other modes of regulation [145]. Profilin-1 not only enhances formin-mediated actin filament elongation, but it also has phosphatidylinositol and microtubule binding capabilities that might serve to modulate both microtubule dynamics and the interplay between microtubules and F-actin important to neuronal GC pathfinding [146,147]. In addition, profilin and capping protein, along with other barbed end binding proteins such as twinfilin, serve to uncap and aid in depolymerization from barbed ends even under assembly conditions, which can aid in the rapid turnover of actin networks [148,149]. Twinfilin may function to either enhance or impede cyclase associated protein (CAP)-enhanced pointed end depolymerization but in a species-specific manner [150]. Filament barbed end association of severing proteins that also cap, such as gelsolin, can be reversed by membrane lipids (e.g., PIP_2_) [145].

**Figure 7:**
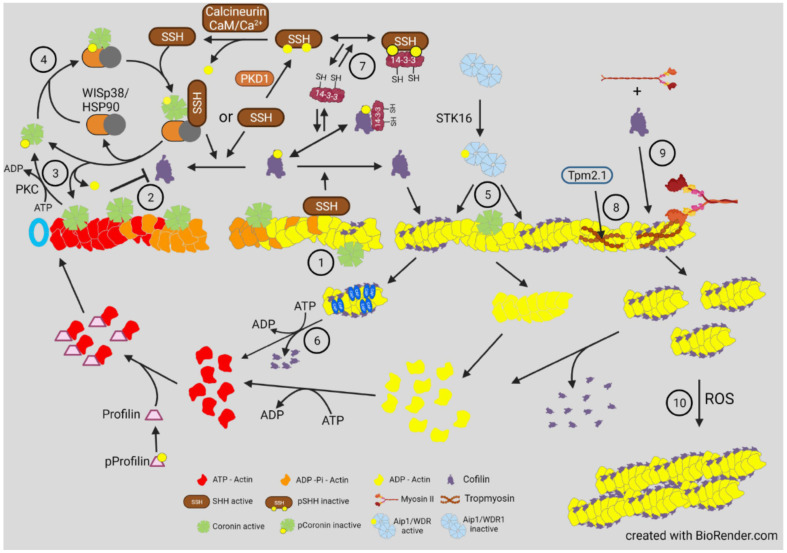
Proteins modulating the turnover of F-actin with cofilin. Coronin’s have multiple roles in actin turnover in terms of being able **①** to recruit cofilin to ADP-actin subunits and **②** to inhibit cofilin binding to ATP/ADP-Pi actin subunits while enhancing Arp2/3 complex binding (not shown) [159]. **③** When phosphorylated by protein kinase C (PKC) on ser2, coronin1B dissociates from filaments and either remains inactive when bound to 14-3-3 until dephosphorylated by SSH [162], or **④** binds a protein scaffold of WISp39/Hsp90, which can then recruit and activate SSH to dephosphorylate cofilin as well as itself [87,89], disrupting the complex and releasing active coronin1B. Dephosphorylated coronin1B can also bind the “aged” ADP-subunits in F-actin, where it serves to recruit cofilin and enhances turnover [160,161]. Aip1, when phosphorylated by the constitutively active STK16, enhances severing **⑤**, and CAP1/2 dimer/oligomers (dark blue) enhance cofilin release and nucleotide exchange on released actin monomers **⑥**. 14-3-3 proteins **⑦** are modulators of phosphoprotein mediated actin dynamics, inhibiting dephosphorylation of several proteins by non-specific phosphatases [81,93,162]. Thus, 14-3-3 proteins are major integrators of phosphoprotein cycles that drive actin dynamics. Different Tpm isoforms **⑧** can inhibit or permit cofilin binding [163]. The Tpm 2.1 isoform allows either cofilin or myoII binding **⑨**, which compete in modulating myoII-dependent contractile processes in cells. **⑩** Fragments of cofilin-saturated F-actin can associate under oxidative conditions to form cofilin-actin rods.

Actin interacting protein 1 (Aip1; also known as WDR1 for its two seven-bladed WD repeats) enhances the severing activity of cofilin [151], primarily at junctions between cofilin-saturated and bare regions of an actin filament [152] (Figure 7). Aip1 activity requires its phosphorylation by a constitutively active membrane-associated kinase, STK16 [153], which may contribute to localized enhancement of cofilin severing depending on the longevity of the phosphorylation. The cyclase-associated proteins (CAP1 and CAP2) aid in complete depolymerization of cofilin-saturated pieces of F-actin by binding as homodimers/oligomers along the filament and releasing separately from different domains cofilin and monomeric actin, the latter of which undergoes nucleotide exchange before release [54,154,155]. CAP1 is essential in mediating cofilin-actin dynamics in growth cones [156]. Deficiency of CAP2 in brains of mice results in a decline in phosphorylated cofilin and unassembled actin and an increase in cofilin-actin aggregates without affecting the levels of other cofilin-phosphoregulatory proteins [157]. In dendritic spines, CAP2 is activated through a sulfhydryl-dependent dimerization, which is deficient in the hippocampus but not in the superior frontal gyrus of both human AD subjects and AD model mice. Cofilin accumulates in these spines, presumably because of decreased CAP2-enhanced turnover, and alters spine plasticity associated with their normal function in memory and learning [158]. The lack of clearance of cofilin will inhibit spine recruitment of drebrin, discussed further below.

Additionally, proteins in the coronin family also help to coordinate and regulate actin filament dynamics by being able to differentially affect newly assembled ATP/ADP-Pi subunits from ADP-subunits (Figure 7). The phosphorylation of coronin-1B on S2 by protein kinase C (PKC) inactivates its F-actin binding, which is restored by its dephosphorylation by SSH1. SSH1 is activated by binding to F-actin or through interaction with a pCoronin/WISp38/Hsp90 complex [89]. When bound to ATP/ADP-Pi actin subunits, coronin1B can activate branching by the Arp2/3 complex and inhibit binding of cofilin, thus, protecting newly assembled F-actin from premature turnover [159]. When bound to ADP-actin subunits, it serves to recruit cofilin and enhances turnover [159,160,161].

Drebrin binds cooperatively to F-actin and increases the helical cross over of the twisted actin chains from 36 nm for naked F-actin to about 40 nm [164], an opposite rotation to that exerted by cofilin, which reduces the filament crossover to 27 nm [6] (Figure 1). Thus, drebrin and cofilin binding are antagonistic, with drebrin stabilizing filaments and cofilin enhancing their turnover. Nowhere is this more evident than in dendritic spines. Both cofilin and drebrin contribute to the synaptic plasticity required for learning and memory by allowing cofilin-dependent spine remodeling and drebrin-dependent spine stabilization in response to presynaptic input [3,115,165,166]. We suggest that neddylation of cofilin, which inhibits its F-actin binding, enhances the ability of drebrin to replace cofilin on spine F-actin during and after spine enlargement, a morphological change that often accompanies the establishment of long-term potentiation (LTP), an oft used electrophysiological correlate of memory. Drebrin also interacts with the microtubule plus-tip binding protein EB3, an interaction that occurs in the proximal region of filopodia where drebrin is bound to F-actin during the formation of a growth cone. The interaction is regulated by phosphorylation of drebrin by cyclin-dependent kinase 5 (cdk5) and is necessary for normal neurite extension [167,168]; this is evidence, again, that supports the necessity of a balance in F-actin stabilization by drebrin and turnover by cofilin. This work also supports the importance of interplay between F-actin stabilizing proteins that can direct microtubule extension through plus-tip complexes along F-actin bundles and F-actin severing and depolymerizing proteins that allow for actin-assembly driven membrane protrusion and the penetration of the growing microtubule tips.

Tropomyosins (Tpms), coil-coiled dimers which assemble head to tail along the sides of F-actin, establish different cellular filament networks [169,170]. There are about 30 isoforms of mammalian Tpms arising from alternative splicing of transcripts from the four Tpm genes [171]. Silencing different isoforms using siRNA suggests that they have non-redundant functions [172]. Skeletal muscle Tpm, now called Tpm1.1 and Tpm1.2, protects F-actin from ADF/cofilin-induced depolymerization in vitro [173], but new isoform-specific functions are being elucidated [163,174]. Specific Tpm isoforms are associated with different F-actin structures in cells, suggesting that they cooperate or compete with other F-actin-binding proteins in defining filament function. Long Tpm isoforms (covering about seven actin subunits in a half twist) generally protect the F-actin from cofilin binding, severing, and depolymerization, but surprisingly they do not enhance myosin II (myoII) ATPase activity and probably do not support myoII contractility associated with its filament binding [163]. The short isoforms, which extend along about 6.5 actin subunits, do not protect filaments from cofilin-induced severing and depolymerization, but these filaments are the ones that enhance myoII ATPase, supporting studies that show competitive cofilin and myoII filament binding [103,104] and enhanced contractile activity in cells silenced for ADF and cofilin expression [175].

The first direct competition between cofilin and Tpm in a specific cellular process was discovered in yeast undergoing cytokinesis [176]. Neuronal effects of Tpms were first reported in mice in which deletion of the Tpm3 gene caused shorter neurites and reduced complexity of branching in hippocampal neurons suggesting that the contribution of Tpm3 isoforms to F-actin stability is required for optimal growth, ostensibly for counteracting cofilin-induced turnover [163,177]. Factors that recruit specific Tpm isoforms to generate the highly diverse Tpm filament populations are not well understood. Actin nucleation by a specific formin, Dia2, recruits specific isoforms of Tpms followed by myoII to form stress fibers [172]. Tropomodulins, F-actin pointed-end binding proteins that also interact with the Tpm, aid in Tpm recognition [178]. However, there are insufficient numbers of tropomodulins to explain by themselves the large diversity in Tpm-F-actin populations. It seems likely that, in addition to these mechanisms, some direct modifications of actin within a filament or an interplay with other F-actin binding partners sort out Tpm isoform specificity. One such possibility is through specific lysine modifications on actin catalyzed by different lysine acetyltransferases (KATs) [179]. Of course, this just pushes our understanding further down the road since the question then becomes “how do KATs recognize the different filaments for modification?”.

An alternative mechanism could be through coordinated and localized translation of actin and its associated proteins. Cytoplasmic β-actins and γ-actins differ in only four amino acids at/near their N-terminus, and yet disruption of the β-actin gene has profound impacts on embryonic development and cell migration, whereas disruption of the γ-actin gene does not. Mice in which changes were made in the β-actin gene to encode a protein with the γ-actin sequence developed normally, demonstrating that it is not the protein sequence but rather the gene sequence that is important [180,181]. The β-actin mRNA contains a 3′UTR zip code which helps direct its localization to the cell periphery for translation, unlike the γ-actin which is translated nearby the cell nucleus [182]. Other modifications are associated with β-actin translation, such as arginylation [183], suggesting that the location where a protein is made is as important to its function as when it is being made. Coordinated translation of families of proteins might explain their mechanisms of sorting easier than a post-translational code, although one does not exclude the other.

Thus, it takes a village of actin regulatory proteins working in a cooperative manner to coordinate the diverse and spatially specific functions required for the vast array of actin-dependent cellular processes, especially in metazoans where tissue specific functions are required. These processes are spatially regulated by the localized components of upstream regulatory pathways that can modulate cofilin directly or modulate one or more of the many other proteins that impact cofilin localization, activation, filament severing, monomer binding, etc., resulting in an exquisite and coordinated control of actin-mediated cellular behavior [184]. Understanding the coordinated regulation of this multitude of proteins has become more complex due to the discovery of additional post-translational modifications of cytoplasmic actin [185], including oxidation/reduction (redox regulation and S-glutathionylation) [123,186], N-terminal arginylation [187], and lysine acetylation [188]. As an example, a specific plasma membrane enzyme called Mical, an NADPH-dependent oxido-reductase [189,190], oxidizes F-actin on specific methionine residues (M44 and M47), which enhances the ability of cofilin to depolymerize these filaments at a faster rate [191]. Whether or not this enhanced actin turnover is the major cellular function of Mical still needs to be established since many other cortical actin-interacting proteins have yet to be studied in the context of oxidized actin. Other NADPH oxidases (NOX) also have a subunit with a defined actin binding site [192].

## 5. Actin Dynamics in Neuritogenesis and Neurite Growth

Brain development is morphologically normal in ADF null (ADF KO) mice [32], but cofilin is required for all aspects of neuronal development. Neuritogenesis, the first morphological steps in neuronal differentiation, does not occur in neuroblasts of ADF KO mice in which cofilin-1 is conditionally knocked out [193]. Although not measured specifically in neuronal progenitor cells, cofilin-2, which is highly upregulated in muscle at later stages of development, is probably not expressed or expressed at very low levels at this stage [30]. What is striking from this study is the presence of membrane parallel actin filaments in electron micrographs of the neuroblasts, suggesting that the formation of neurites is regulated by the need for cofilin-induced disassembly of the membrane proximal F-actin, such as what has been described above for polarizing fibroblasts [101]. The breakdown of the membrane proximal F-actin by cofilin is followed by nucleated assembly of actin filaments perpendicular to the membrane, driving filopodial and lamellipodial protrusions that emerge as the GC.

De novo neurite outgrowth and regeneration in adult neurons also requires actin turnover mediated by cofilin [24]. Two cofilin activities can be distinguished based on site directed mutants: S94D can depolymerize but not sever filaments and Y82F can sever but not enhance depolymerization [194,195]. When these mutants are expressed individually in an adult axon regeneration system in which endogenous ADF and cofilin are silenced, severing is the essential activity for regeneration [24].

Although the outgrowth and migration of the GC is reasonably well understood at the molecular level [196], the question of how cofilin regulation fits into the guidance of growth cones has been more controversial. Ratio imaging of immunofluorescence images of stained total cofilin to phospho-ADF/cofilin in GCs showed higher levels of active cofilin along the region of the growth cone opposite to the side in contact with a repulsive guidance cue (aggrecan) [197]. In chick retinal or dorsal root ganglion neurons, which express ADF and not cofilin-1, an attractive turning response was observed to a gradient of a membrane permeable source of either WT or S3A ADF, but not S3E, with increased filament barbed ends on the growth cone side turning toward the source of the ADF [198], suggesting that enhanced severing and generation of new barbed ends might be sufficient for setting the direction of protrusion and growth.

However, opposite results for phospho-cofilin distribution were observed in *Xenopus laevis* spinal neurons responding in culture to a gradient of bone morphogenic protein 7 (BMP7), an attractant for these growth cones in their early developmental stage (0–8 h) [199]. During this period, the BMP7/receptor activated LIMK1 was essential in turning and resulted in increased phospho-cofilin on the growth cone side in the direction of turning. Surprisingly, the response to BMP-7 reversed in neurons between 8 and 20 h in culture, and BMP7 became a repulsive cue due to expression of a transient receptor potential channel (TRPC) that responded to BMP7 by allowing calcium influx, which activated calcineurin, a Ca^2+^-calmodulin dependent phosphatase previously shown to activate SSH in neurons [98,199] (Figure 5). The distribution of cofilin activity across the growth cone was reversed during repulsion, which was blocked with calcineurin inhibitors. The timing of the switch between attraction and repulsion in vitro correlates with ventral projections of commissural neurons in vivo, which first benefitted from the attraction to the BMP7 producing cells and then by repulsion that aids in their growth past the site of BMP7 release. Taken together, these findings suggest that it is more than just a bias in activated cofilin that is required for the dynamic regulation of actin. It is possible that a decrease in activated cofilin might enhance its severing activity, which is optimal at about 10 nM on isolated filaments in vitro [8]. However, as shown in the bead experiment described in Figure 2, there is likely a broad range of cofilin concentrations (nM to μM) that can support steady state dynamics of actin [51]. In cells, cofilin phosphocycling might be more important than the amount of cofilin that is in the dephosphorylated pool. Alternatively, other proteins that enhance severing and turnover of cofilin-bound actin discussed above (Figure 7) may also have gradients of activity across a growth cone and perhaps may not require any changes in phosphorylated cofilin to control turning response.

## 6. Actin Dynamics in Neurite Consolidation and Branching

Temporal regulation of actin dynamics is also important in the consolidation process by which the neurite shaft forms behind the extending growth cone and in neurite branching. A current theory of consolidation is that it requires repressing protrusive activity stimulated by cortactin, an activator of the Arp2/3 complex, that helps form branched actin networks associated with new protrusions [200]. Cortactin is also an enhancer of deposition of extracellular matrix material stimulating branching [201]. Cortactin is highly sensitive to degradation by calpain, a calcium-activated protease within neurite shafts. By degrading cortactin, calpain limits new protrusions. The branching of more mature neurites probably occurs by reversal of this pathway through inhibition of calpain by phosphorylation catalyzed by the cyclic AMP-dependent protein kinase A (PKA). This pathway likely explains the branching at sites of contact between neurites and neurotrophin-coated beads that locally activate adenylate cyclase to produce cAMP [202]. Neurite branching also requires septins, a family of GTP-binding proteins that can assemble into filaments, rings, and mesh works and which then recruit cortactin to new sites of collateral branch formation [203]. Cortactin also aids septins in directing neurite microtubules toward the site of protrusion where branched actin networks assemble on the tip of the microtubule through the activity of the adenomatous polyposis coli (APC) protein [204]. The penetration of a protrusion by a microtubule is required for the delivery of mitochondria and the development of the GC for neurite branch elongation [203,205].

Significant branching of primary neurites also occurs during early stages of outgrowth in cultured hippocampal neurons. The consolidation phase of the neurite at the base of the growth cone is often broken by the transit of new lamellipodial-like processes (waves), usually starting at the soma and migrating along the neurite [206,207]. During the period of axonogenesis, waves increase in frequency along the future axon [208,209]. On some substrates, neurites elongate faster when the wave reaches the growth cone, although myosin II-induced alterations in growth cone shape may result in an apparent surge forward [210]. During early neurite extension in cultured hippocampal neurons, waves move into and expand the tip of a filopodium and form a new growth cone establishing a branch off the neurite. The ability of the waves to extend down the neurite depends upon their binding to substrate through receptors, such as the L1-cell adhesion molecule (L1-CAM), which couples with cortactin and the F-actin network that is undergoing retrograde flow (treadmilling) with the forward protrusion of the membrane driven by actin assembly. This “molecular clutch” is provided by shootin-1b [211]. Actomyosin and the microtubule motor dynein also contribute to the forces for neurite elongation [212]. Waves transport actin toward the neurite tip probably by a preferential reutilization of actin subunits disassembled by cofilin from treadmilling filaments and is, to date, one of only two demonstrable mechanisms for neurite actin transport, the other being similar with nucleating hot spots that result in a biased anterograde elongation of filaments within neurites [213].

Long-term stabilization of the neurite shaft comes from membrane associated actin-rings that are spaced at about 190 nm by spectrin tetramers [214,215]. Although initially thought to be short, capped filaments, recent studies using platinum replicate electron microscopy and super-resolution microscopy show that filaments in the rings are composed of two long intertwined F-actins connected by a dense meshwork of aligned spectrins [216]. The rings form earlier in a presumptive axon than in a dendrite and extend throughout the axon shaft, whereas they are not as complete throughout its length in a dendrite [217]. Rings compartmentalize the membrane and restrict diffusion of lipids within the axon initial segment [218], but they do not form early enough to explain neurite consolidation. Rings associate with myosin II, which has a scaffolding and/or contractile role that can alter axon electrophysiology [219]. The actin ring network in axons is more stable with respect to remodeling than in dendrites [220,221], but the rings can expand and contract during cargo passage. Although the effects of cofilin on ring stability and turnover have yet to be reported, it is possible that cofilin on its own may not directly interact with the actin in rings given their structure and extensive spectrin cross-linking and myoII binding.

If cofilin is unable to disassemble ring actin, what other proteins might do so? Calpain, the cortactin-degrading protein in shaft consolidation, also degrades ring structures during a Ca^2+^-dependent degenerative response [222], although a milder Ca^2+^-induced F-actin disassembly might be provided by gelsolin, a Ca^2+^-dependent severing and barbed-end capping protein that is expressed in neurons [223]. Alternatively, an F-actin severing activity has been identified as a function of a large (250 kDa) multidomain protein containing a leucine-rich repeat and kinase domains called Lrrk2. As a monomer or dimer, Lrrk2 severs F-actin in vitro but loses this activity when further oligomerized [224]. Its severing activity is of interest because mutations in Lrrk2 that affect severing are the most common mutations found in familial Parkinson’s disease (PD). Furthermore, Lrrk2 interacts with α-synuclein, resulting in Lrrk2 oligomerization, loss of severing activity, and enhanced stability of F-actin in *Drosophila* models of PD. These can be partially reversed in flies by overexpressing *Drosophila* cofilin (twinstar). The decreased turnover of F-actin in neurons with mutant Lrrk2 results in the mis-localization of dynamin-related protein 1 (Drp1) and subsequent mitochondrial elongation and dysfunction, discussed further below. The role of α-synuclein, the major amyloid component of Lewy bodies, in dysregulating Lrrk2 is of particular interest for understanding dementia, which occurs in a significant number of PD patients who have triplication of the α-synuclein gene [225].

## 7. Cofilin Organelle Localization and Functional Consequences

### 7.1. Nucleus

The ability of cofilin to accumulate with actin in the cell nucleus as rod-shaped bundles of ~10 nm filaments was first observed in cultured cells stressed by treatment with 10% DMSO or by heat shock [226]. Rods immunostain for actin but do not stain with fluorescent phalloidin, demonstrating that they do not contain “typical” F-actin. Another protein associated with and required for nuclear rods induced by 10% DMSO is an enhancer of cofilin-actin severing, Aip1/WDR1 [227], suggesting that nuclear rods might arise from fragments of cofilin-saturated nuclear F-actin. Although use of 10% DMSO for rod-induction may seem irrelevant to biology, it is commonly used as the major cryoprotectant in cell freezing for long-term storage in liquid nitrogen. Perhaps sequestering some actin into nuclear rods reduces cortical actin networks and provides more elasticity to the plasma membrane that helps keep it intact during cytoplasmic expansion due to freezing.

Cofilin contains a nuclear localization sequence (NLS; ^30^KKRKK^34^) similar to that of the SV40 large T-antigen [228]. Additional residues comprising a bipartite NLS have since been identified [229] (Figure 4). All three ADF/cofilin isoforms have been identified in nuclear rods from different cell types [226,230,231].

**Figure 8:**
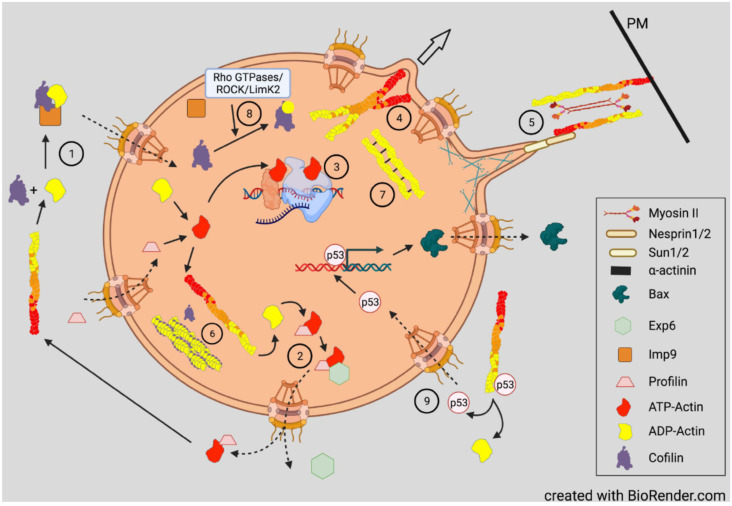
Nuclear uptake and nuclear functions of cofilin. Cofilin can chaperone actin transport into the nucleus **①** via Importin 9, probably as ADP-actin to which it has much higher affinity than to ATP-actin. Export of actin **②** is via profilin-actin-Exportin 6. **③** Nuclear actin is a required subunit in RNA polymerases and chromatin remodeling factors [240]. Forces within the nucleus that result in nuclear envelope protrusions **④** are driven by actin assembly [241], but strong cytoplasmic forces **⑤** by filaments linking the nuclear matrix through the nuclear envelope via SUN1/2 and nesprin1/2 also result in nuclear deformations that are controlled by cofilin competing with cytoplasmic myoII [176]. Actin rods in the nucleus **⑥** form under stress and may contain cofilin (heat shock stress) as well as being formed from αlpha-actinin and actin **⑦**, especially prevalent in some muscle diseases [242]. α-Actinin-4 mediates gene expression for proliferation by binding beta-catenin, which is an activator of the wnt signaling pathway for cell proliferation. Rods that form under stress tie up the α-actinin, blocking this cell proliferation pathway. Thus, rod formation might be a rapid and efficient method for sequestering proteins in response to stress. **⑧** Nuclear Rho GTPases can signal via ROCK/PAKs to LIMK2 to inhibit proliferation (a decline in LIMK 2 enhances tumor progression via beta-catenin and wnt signaling). Active LIMK2 causes cell cycle arrest at the G1/S transition. Nuclear uptake **⑨** of p53, a tumor suppressing protein that stimulates the production of BAX and other proapoptotic genes, is regulated in part by cytoplasmic F-actin [243,244] and cofilin-mediated depolymerization aids in p53 nuclear translocation. Further details on nuclear actin may be found in [240,241].

Cofilin transports a complex of monomeric actin and importin 9 into the nucleus via the nuclear pore complex (NPC) [232,233]. Actin export from the nucleus through the NPC is mediated by profilin and exportin 6 [234]. The dynamics of nuclear F-actin are mediated at least in part by a pool of nuclear cofilin, which increases with enhanced transport of G-actin [229,235]. Monomeric nuclear actin fills many roles in transcriptional regulation, first suggested in 1984 [236], including serving as a component of all three RNA polymerases [237] and in many chromatin-remodeling complexes [238]. Alterations in nuclear transport of actin has major effects on responses to growth stimulatory pathways, such as that activated by serum response factor [239], and on the overall transcriptome [33,240] (Figure 8).

An optogenetic technique controlling the exposure of a nuclear export signal on cofilin-1 was used to demonstrate that a cofilin-regulated transient pool of nuclear F-actin is required for chromatin decondensation following cell exit from mitosis into G1 [241]. The transient F-actin pool assembles at the nuclear envelope and results in protrusions and nuclear volume expansion as well as in chromatin organization in daughter nuclei. Nuclear cofilin-1 inactivation by phosphorylation occurs concomitantly with transition into G1 as nuclear F-actin increases. However, nuclear actin assembly promoted by G-protein coupled receptor (GPCR)-mediated calcium signaling via the formin INF2 [245] also enhances nuclear protrusions and volume expansion in G1 that require filament bundling by the F-actin cross-linking protein α-actinin 4 (ACTN4) [246], a mediator of endothelial mesenchymal transformation, tumorigenesis, and cancer metastasis [247,248,249,250]. Significantly regarding its intranuclear functions, α-actinin 4 (along with α-actinin 2) is found as a component of intranuclear rods that are devoid of cofilin, but which form in response to the expression of a skeletal muscle α-actin mutation (V163L) that causes intranuclear rod myopathy [251], suggesting that rod formation is a conserved mechanism for sequestering potentially harmful proteins and is part of the actin stress response. One hypothesis is that chromatin remodeling and transcription processes utilize monomeric actin as machinery components but may also utilize the dynamic nuclear actin assembly and can be slowed under stress by sequestering monomeric actin in the nucleus [240]. Live imaging of actin^(V163L)^ -EGFP incorporated into the intranuclear rods in fibroblasts demonstrated significant rod bending and torsional motion, with one rod turning almost 90 degrees in 6 s without an apparent change in nuclear shape, suggesting strong intranuclear contractile forces. Nuclear rods are also found in some nemaline myopathies [252].

Phospho-regulation of cofilin in the nucleus is not well understood. A genome wide siRNA screen in Drosophila for modulators of nuclear actin import or polymerization identified several cofilin regulatory proteins that have mammalian homologs [253]. One of these repressed the expression of a cofilin kinase. In mammalian cells, both LIMK 1 and LIMK 2 are found in the nucleus. As in cytoplasmic signaling, Rho GTPases also function in nuclear signaling, especially in DNA damage/repair, a process in which cofilin has also been implicated [254], so a pathway for LIMK phospho-activation via a Rho GTPase effector such as ROCK/PAK likely mediates the aspects of this process [255] (Figure 8). Surprisingly, evidence suggests LIMK1 and LIMK2 affect nuclear function but in opposite directions, with an imbalance proposed to affect cell proliferation and metastasis in some cancers [256,257]. A decline in nuclear LIMK2 enhances a tumor progression signaling pathway mediated by β-catenin through an activated Wnt signaling pathway. LIMK2 inhibits cell proliferation by cell cycle arrest at the G1/S transition [257], but whether this is mediated by cofilin needs to be substantiated as another LIMK2 substrate was identified as an E3 ubiquitin ligase adaptor called SPOP [258]. LIMK2 phosphorylates SPOP on three sites that enhance its degradation, but SPOP opposingly promotes LIMK2 ubiquitinylation and degradation, creating an interesting feedback regulatory cycle [259]. Additionally, T505, the phospho-activating residue in LIMK2, is one of a few select sites targeted by the mitotic and cell cycle regulator, Aurora A kinase, which also serves as a substrate for LIMK2 in a positive activation loop associated with mitotic regulation [260]. Interestingly, Aurora A phosphorylation of LIMK2 also targets ser283, which inhibits nuclear uptake of LIMK2 [257]. The combination of the effects of SPOP and Aurora A kinase on nuclear activity of LIMK2 may affect cofilin’s nuclear function, but a well-defined cofilin-dependent process in the nucleus is needed to address this issue.

Studies of nuclear actin dynamics suggest sites of assembly at the nuclear envelope that can expand the nuclear membrane and alter nuclear shape [241] (Figure 8). However, cytoplasmic actin filaments are linked to nesprin 1/2, a nuclear outer membrane component of a transnuclear outer/inner membrane complex that links the nuclear lamina, an intermediate filament system underlying the inner nuclear envelope, with the cytoplasmic cytoskeleton. In the absence of ADF/cofilin to compete with myosin II for relieving tension, long extensions of the nuclear envelope with bound chromatin are pulled into the surface blebs of the plasma membrane [176]. Such architectural restructuring of the nuclear envelope results in changes in patterns of histone modifications that distinguish condensed (transcriptionally inactive) heterochromatin versus euchromatin. The extent to which cytoplasmic versus nuclear cofilin/actin pools modulate transcription through chromatin architectural changes requires further study. Such changes may impact the early stages of neuronal birth in the brain during which neurons arise from a progenitor (radial glia) cell. The nucleus of the progenitor cell translocates from the subventricular zone to the ventricular zone where an asymmetric division results in one neuron and one progenitor cell. Both microtubule-dependent and actin-dependent motors play important roles in this asymmetric division [261]. It would be of interest to know if the cytoskeletal organization and tension on the nuclear envelope before or after the asymmetric division affects chromatin decompaction and cell fate-specific gene expression. Given the recently developed novel methods for studying single gene transcription, nuclear exit of the mRNA, and its cytoplasmic translation, the answers to questions concerning cytoplasmic forces on chromatin structure and gene expression are not far off [262].

### 7.2. Mitochondria

**Figure 9:**
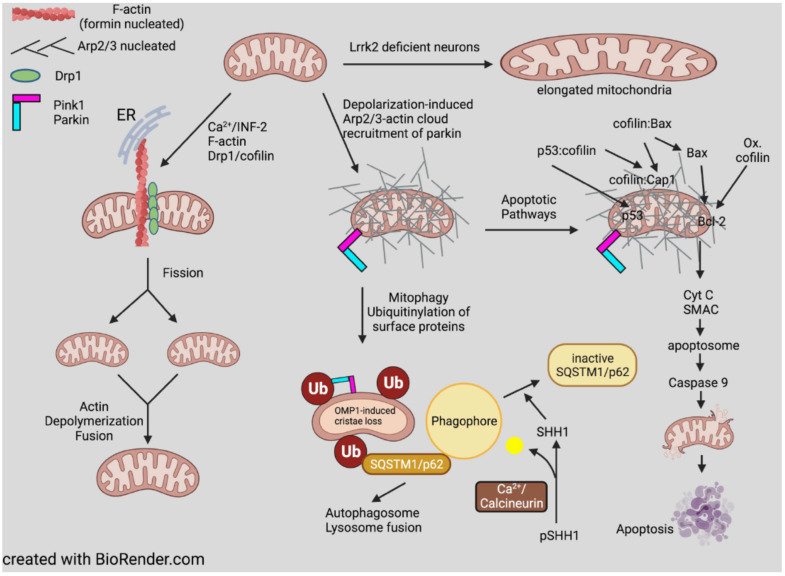
Role of actin in mitochondrial fission/fusion, mitophagy, and apoptosis. Cofilin aids in the restructuring of INF2-nucleated F-actin along ER membrane and recruitment of Drp1 to stimulate mitochondrial fission initiated by a local increase in cytoplasmic Ca^2+^ [264]. Drp1 interacts with mitochondrial outer membrane proteins to stimulate a constrictive force around the mitochondrion [265,266]. Dynamic actin along with myoII is required for the proper recruitment of Drp1. Excess actin stabilization, such as what occurs in neurons defective in the actin severing protein Lrrk2, results in Drp1 mis-localization and mitochondrial elongation and may contribute to mitochondrial defects observed in neurodegenerative diseases [224,267]. Disassembly of the actin occurs during mitochondrial fusion allowing mixing of the contents to maintain healthier mitochondria. Extended mitochondrial depolarization results in mitophagy, the engulfment and lysosomal degradation of mitochondria [268]. Mitophagy is independent of Drp1 and is initiated by local activation of the Arp2/3 complex and formation of an actin cloud (gray filaments) surrounding the mitochondrion [264]. Activation of a mitochondrial outer membrane protease (OMP1) initiates the loss of cristae and the circularization of the inner mitochondrial membrane, independent from cytoplasmic actin-mediated events. The engulfment of mitochondria by the ER-derived phagophore is dependent on the autophagy cargo receptor, SQSTM1/p62, which recognizes ubiquitinylated surface proteins [269], the formation of which is largely the responsibility of the E3 ubiquitin ligase, Parkin, which is recruited to the mitochondrial surface by the molecular sensor for damaged mitochondria, PINK1 [268]. SQSTM1/p62 is activated by multiple kinases through S403 phosphorylation. Dephosphorylation of SQSTM1/p62 is catalyzed by SSH1, which inhibits mitophagy [270] and might enhance the mitochondrial apoptotic pathway through enhanced release of proapoptotic signals. This can be triggered by oxidized cofilin [63,64] and, in some cell types, by uptake of cofilin complexed with the tumor suppressor (proapoptotic) protein, p53 [244]. Additionally, cofilin can bind to Bax, an activator of the proapoptotic Bcl-2 which permeabilizes the outer membrane to release of apotosome activators such as cytochrome c. Cofilin could serve to translocate Bax to the F-actin cloud surrounding mitochondria [271], where Bax can be released, perhaps through cofilin binding to CAP1, which has both mitochondrial and cofilin binding domains and is necessary for cofilin-mediated apoptosis [268,272,273]. Cofilin binding to p53 targets mitochondria, where it might have a direct pathway for initiating apoptosis separate from the nuclear effects described in Figure 8 [244]. More details can be found in [2].

Mitochondria, often major sources of ROS production in stressed cells, can undergo both fission and fusion to modulate their numbers and cellular distribution; abnormalities in their dynamics and transport are linked to many neurodegenerative diseases [263]. The fission and fusion processes allow the mixing of mitochondrial contents. Removal of damaged mitochondria by mitophagy is also a normal process in healthy cells, but mitochondria also serve as a sensor to trigger cell death through apoptosis (Figure 9).

A balance in fission/fusion is required to maintain mitochondrial numbers but is subjected to shifts that are dependent on actin dynamics [2]. What is of interest is that the actin reorganization associated with Drp1-induced mitochondrial fission is cofilin-specific, as ADF cannot replace cofilin in this process [274]. Maintaining a healthy mitochondrial network depends on a balance between mitophagy to remove spent mitochondria and fission/fusion events to generate more mitochondria. Such a balance is extremely important in neuronal health because, in axons, these processes are occurring often hundreds or thousands of cell body diameters away from the soma.

Staurosporine-induced apoptosis is inhibited by silencing cofilin or by overexpressing the inactive phosphomimetic cofilin S3D [63]. What is also mediated by cofilin is apoptosis induced by oxidative stress during which M115 of cofilin is converted to a sulfoxide, eliminating cofilin-actin binding [120]. Cofilin also undergoes oxidation of its four cysteines to form one or two intramolecular disulfides necessary for its accumulation at mitochondria [64]. If apoptotic progression arises from cofilin localization of Bax to the actin cloud surrounding mitochondria, reversal of the M115 oxidation by sulfoxide reductase would need to occur. However, if the cofilin-Bax complex is recognized by CAP1, perhaps this interaction would result in Bax release at the mitochondrial surface regardless of the ability of cofilin to bind actin. The functioning of SSH1 as both a cofilin activator and an inhibitor of mitophagy suggests that both processes contribute to the neuronal degenerative response, one through enhanced cofilin-actin rod formation and the other through inhibiting the clearance of autophagic cargo [270].

With the exception of phosphorylation and oxidation, post-translational modifications of cofilin have not yet been studied in the context of mitochondrial targeting or apoptosis. However, another post-translational pathway in which actin subunits are directly modified conceptually expands new models for actin-dependent processes. Acetylation of lysine residues (K50 or K61) on ATP-actin modulates its polymerization by forming an inhibitory complex with CAP1 or CAP2 to bind the formin INF2 and blocks its ability to elongate actin filaments [188]. Protein acetylation on lysines was first discovered on histones [274] and the enzymes catalyzing the acetylation/deacetylation (histone acetyl transferase, HAT; histone deacetylase, HDAC) were discovered the following decade [275,276] and have become targets in cancer therapy [277]. Many non-histone substrates, including microtubules, have been subsequently identified, and the enzymes are now sometimes classified as KATs (lysine acetyl transferases) and KDACs (lysine deacetylases). One KAT family, sirtuins, was previously mentioned as NAD+-dependent modifiers of Hsp90, resulting in cofilin dephosphorylation, presumably by the release of CIN. A specific KAT acetylates K50 and K61 on actin [188]. More than 20 different KATs have been identified based on sequence homology [179], suggesting that KAT/KDAC mediated modifications of F-actin might form an “actin code” that can modulate actin assembly pools differentially. HDAC6 plays an important role in neuronal polarity and, in AD, possibly by increasing Hsp90 acetylation and/or tau phosphorylation [278,279]. However, Hsp90 hyperacetylation might affect binding and activity of CIN [96], so the role of HDAC6 in actin dynamics needs further study. Determining if different actin KATs modify filaments in ways that allow differential binding of specific isoforms of Tpms, coronins, or ADF/cofilins is also of importance. There are 19 lysines on cytoplasmic actins, at least seven of which have been reported to be acetylated [179].

## 8. Cofilin-Actin Rods in Neurodegenerative Diseases and Disorders

### 8.1. Rod Induction and Visualization

The formation of cytoplasmic rod-shaped bundles containing cofilin and actin have been studied in neurons: (1) overexpressing or acutely activating (dephosphorylating) cofilin; (2) exposed to energetic, oxidative or excitotoxic stress including hypoxia/ischemia; and (3) exposed to factors associated with progressive cognitive decline in age-related neurodegenerative diseases. Rods form within neurites, but only rarely in the cell soma, in response to energetic and oxidative stress. For the several cofilin antibodies that we have utilized, visualization of rods by immunostaining following aldehyde fixation requires permeabilization with 100% methanol (−20 °C) rather than with a non-ionic detergent [42]. Rods visualized by actin immunostaining after using non-ionic detergent permeabilization do not stain or stain weakly with fluorescent phalloidin, which stains other F-actin structures in the same cell, suggesting actin in rods is saturated with cofilin, which eliminates phalloidin binding [6,42]. Rods do not contain S3 phosphorylated cofilin [280,281].

### 8.2. Rod Structure and Composition

In aldehyde fixed, ATP-depleted cells, rods are composed of wavy bundles of ~10 nm filaments for which its lengths are difficult to follow in transmission electron microscope (TEM) tomograms generated from tilted images of thick sections. However, in cells that are plunge frozen and freeze substituted, rods contain straighter parallel filaments in which the individual lengths run from fragments of only a few actin subunits to filaments of over 1 μm, with a median length of 200 nm (80 actin subunits) [65], suggesting that they form from cofilin-saturated fragments (Figure 1).

Rods induced by energy depletion have been isolated from both cortical neurons and non-neuronal cells and contain the two cytoplasmic (β and γ) actin isoforms and ADF/cofilin in a ratio between total actin and total ADF/cofilin of 1:1. ADF and cofilin in rods are present at about their same expression level as within the cell type from which they are isolated [65,231]. Other components identified by mass spectrometry in non-neuronal rod preparations include peroxiredoxin 1, annexin A2, hsp60, and 14-3-3 (isoform unidentified); of these, only 14-3-3 was identified in rods by immunostaining, and it accumulated in later stages of rod development. A phospho-tau antibody immunostained rods in mouse neurons, even those formed in neurons from a tau knock-out mouse [282,283], but it did not stain rods in human AD brain in which it was specific for phospho-tau. The protein it recognized in mouse brain remains unidentified.

Rods have been formed in vitro from equimolar mixtures of either ADF/actin or cofilin/actin [231]. Rods isolated from neurons and non-neuronal cells behaved similarly, disappearing after 10 min exposure to 0.05% Triton X-100, declining by 50% in 0.5 M NaCl, but stable in 5 mM of either DTT, ATP, or CaCl_2_ added to the rod isolation buffer. Most cofilin exists as a disulfide cross-linked dimer in neuronal rods isolated after cell lysis into a solution in which free sulfhydryl groups were rapidly blocked with iodoacetamide. Cysteines C39 and C147 were implicated as forming the intermolecular disulfide. No cross-linked cofilin-actin species were observed [65].

Covalently cross-linked cofilin-actin complexes do occur in nuclear rods that are associated with a cell model for Huntington’s disease (HD) [284]. HD arises from expansion of a CAG codon in the huntingtin gene, encoding long repeats of glutamine (polyQ) in the expressed protein. Nuclear rods induced by heat shock or 10% DMSO in a striatal neuron-derived cell line expressing either mutant (Q111) or normal (Q7) huntingtin protein [284] were not only immunostained for cofilin and actin but also for huntingtin protein. Cells expressing WT (Q7) huntingtin form numerous short rods, almost all of which disappeared within 3 h after recovery from heat shock, whereas cells expressing the huntingtin (Q111) had fewer and longer rods, which persisted in over 35% of cells at 24 h post heat shock. The persistence correlates with the formation of covalent cofilin-actin complexes formed by tissue transglutaminase 2 (TG2), which is recruited by the mutant huntingtin to nuclear rods. Thus, multiple mechanisms exist for generating cross-linked complexes in rods, some of which are much less reversible.

Friedreich’s ataxia (FRDA) is a peripheral neuropathy in which there is an early loss of sensory dorsal root ganglion neurons. It arises from another triplet codon expansion, this time a GAA expansion encoding glutamate, in the gene for a mitochondrial protein called frataxin, causing its loss of function. In the frataxin YG8R mouse model of FRDA, there is a demonstrable inhibition of axonal transport, and neurons cultured from this mouse have small, abnormal growth cones [285]. Increased expression of CIN resulting in hyperactivation of cofilin was also demonstrated. Unfortunately, rod formation was not examined, although an increase in phalloidin staining (increased F-actin/G-actin ratio) occurred in DRG neurons from the YG8R mouse compared to the WT control after non-ionic detergent permeabilization.

Cofilin-actin rods have been found in motor neurons in spinal muscle atrophy (SMA), a disease initiated by the reduction in a protein named Survival of Motor Neuron (SMN) [286]. Rods accumulate a cytoplasmic cleavage product of the axonal growth guidance receptor PlexinD1 and are proposed as a sink for sequestering this fragment in dysregulating receptor signaling. A motor neuron-like cell model of SMA has allowed for the enrichment and crude fractionation of rods and characterization of proteins associated with them [287]. Profilin-2, a phospho-regulated modulator of actin assembly [288], but not the more prevalent profilin-1 was found as a rod component in SMN-depleted cells. Phospho-inactivation of profilin-2 on S137 reduced its ability to bind actin monomers. The overexpression of the S137A mutant reduced the number of cells with rods, suggesting that profilin-2 sequestering of G-actin or enhancing nucleotide exchange to ATP-actin decreased the cofilin-ADP-actin pool for rod incorporation.

### 8.3. Rods Induced by Excitotoxicity

Glutamate (100–150 μM) induces cofilin-actin rods within 30–60 min in rodent hippocampal neurons cultured 5–6 DIV [42]. The rod response is mimicked by a specific agonist to the subclass of glutamate receptors responsive to AMPA (α-amino-3-hydroxy-5-methyl-4-isoxazolepropionic acid) [289]. More mature neurons, 10–14 DIV, utilized both AMPAR and the calcium-permissive NMDA (N-methyl-d-aspartate) receptors for rod induction, which also occurs during a similar time course [290]. SSH1 dephosphorylation by calcineurin may be the mechanism activating cofilin [98].

### 8.4. Rods Induced by Ischemia

The rapidity of rod formation in energetically stressed and glutamate-treated neurons and in brain slices undergoing oxygen deprivation [42,76] resulted in studies on rod formation in ischemic brain injury (stroke). Four different mouse models of brain ischemia were examined, each initiating rod formation but with differing patterns [291]. In all models, rods formed exclusively in neuronal processes. In some models, such as middle cerebral artery occlusion (MCAO), rod scores (rod area as a percent of total field area) that resulted immediately after the ischemic event were low but increased over 24 h following the start of reperfusion. In other models, rods were elevated at the start of reperfusion and declined over 24 h, but the time course differed somewhat between brain regions affected by ischemia. Photothrombic lesions produced the most localized ischemic event; rods started forming within 1 h and reached their maximum at 24 h, localizing within the distal edge of the infarct region and slightly into the surrounding non-ischemic tissue but never within the core.

A detailed study using the MCAO reperfusion model was performed in rats [292]. Cofilin-actin rod formation correlated with a decline in dendritic staining for MAP2 in the affected area. Regions with rods showed almost complete disappearance of functional mitochondria, and synaptic activity declined in the neurons with rods. Treatments that promoted cofilin phosphorylation, e.g., with the Rho GTPase activator CN03 which activated the ROCK-LIMK pathway, reduced rod formation whereas enhancing cofilin dephosphorylation with the ROCK inhibitor Y-27632 increased rods and exacerbated synapse loss. LIMK overexpression reduces rod formation, protects synapses, reduces MAP2 degradation, and attenuates cofilin-mediated apoptosis, increasing neuronal survival following stroke [293] and supporting the finding that decreased cofilin activity is beneficial in recovery from strokes.

Ischemic tissue damage is not restricted to neurons. Ischemic kidney injury is a major cause of death after traffic accidents. Induced ischemia followed by reperfusion in rodent kidney results in the loss of apical membrane from proximal tubule cells. Apical membrane microvilli are disassembled with sluffing from the cell of membrane vesicles, which appear in the urine, some of which contain cofilin-actin rods [294].

### 8.5. Rod Pathology in Age-Related Neurodegenerative Diseases

AD, the first age-related dementia to be recognized [295] is defined by its pathology of extracellular amyloid plaques, containing fibrils of the amyloid-β (Aβ) peptides cleaved from the single pass transmembrane amyloid precursor protein (APP), and intracellular neurofibrillary tangles, containing hyperphosphorylated tau which is a microtubule binding and stabilizing protein with additional functions [296]. Most familial forms of AD arise from mutations in APP, in the pathway of Aβ production, or in reduced Aβ clearance, resulting in its focus over decades as a causative factor in AD even though familial cases of AD account for only a few percent. Aβ is cleaved from APP by β-secretase at D672 and by γ-secretase (presenilins) within the membrane spanning domain at about residue 713 to provide Aβ peptides commonly ranging from 39 to 42 amino acids depending on the intramembrane cleavage site. Aβ self-assembles into oligomers progressing to insoluble fibers, which deposit in extracellular senile plaques. Plaques are neither highly correlative nor necessary for cognitive decline. Only small dimers/oligomers of soluble Aβ are present in the brains of subjects with a familial Aβ deletion (ΔE22) (Osaka mutation, Δ693 in APP) [297], but no plaques are present even though tau pathology and cognitive decline follow the same time course as in AD patients with significant plaque burden [298].

Cofilin-actin rod pathology, significantly more prevalent in brains of human AD subjects compared to controls [42,299], is found in brains of AD but not WT mice [99]. Defects in axonal transport characterize early stages of many neurodegenerative diseases, and rod formation impairs vesicular transport [280,300], which can be regained after reversal of rods by the removal of the inducer [281,289]. Observation of rods in live cells utilizes a genetically encoded rod reporter made from a point mutation in cofilin (R21Q) that allows rod association without inducing rod formation through its overexpression. Rod reversal to 50% of maximum rod area occurred within 35–45 min with a resumption in vesicle trafficking taking about 3 h. Since hyperactive cofilin affects microtubule stability and tau binding [1], disruption of transport, which affects neurites with no visible rods, probably results from a deficit in microtubule-mediated vesicle motility rather than a physical blockage by the rod.

Rods are associated with several cognitive disorders and share a common pathway for their formation in the subpopulation of neurons affected. Rod formation in culture systems is causative of synaptic deficits, but rods only form in a subset of hippocampal neurons in response to disease-related degenerative factors. However, only a subpopulation of neurons in the hippocampus undergo loss of dendritic spines in a sleep deprivation model in which cognitive deficits are significant [301,302]. Given the importance of cofilin in modulating dynamic actin processes for synaptic function, it is not yet possible to conclude that rods per se are causative with respect to the cognitive dysfunctions that arise.

Overexpression or hyperactivation of cofilin in neurons induces rods. Acute hyperactivation is observed in hippocampal neurons in response to the release of CIN from hsp90 [94]. In AD mice, reducing total cofilin expression, blocking cofilin activation by reducing expression of an upstream component (RanBP9) of SSH1 activation, or administering cell-penetrating peptides that inhibit cofilin dephosphorylation all reduce rod pathology as well as reverse cognitive deficits [99,303,304]. In cultured hippocampal slices and dissociated neurons, rods are induced by various forms of amyloid-β (Aβ). Aβ oligomers (Aβo) are the most potent rod-inducing form of synthetic Aβ, but rods form more slowly compared to glutamate or ATP-depletion (T_½_ ~6 hrs to maximum response) and form only in a subset (~20%) of rat (E18) or mouse (E16.5) DIV 6–7 hippocampal neurons. A secreted form of Aβ [305], containing mostly Aβ dimers and trimers (Aβd/t) which are the most prevalent soluble pools of Aβ in human AD brain [306,307,308], is 4000-fold more potent in rod induction (EC_50_ ~0.15 nM) compared to synthetic Aβo [309], but the responding cell population does not change. Aβd/t-induced rod formation in rodent hippocampal slices is most pronounced in the dentate gyrus [76]. The percent of neurons forming rods in response to Aβd/t is reduced by treatments enhancing cofilin phosphorylation (e.g., RhoA activation) and is increased by treatments enhancing dephosphorylation [1,309].

Rods are also induced in rodent neuronal cultures by proinflammatory cytokines [281] and by the HIV envelope protein gp120 [310]), which is the proposed initiator of HIV-associated neurocognitive disorder (HAND) [311]. The lack of any additive effect of these inducers when used in combination suggests that the identical 20% subpopulation of neurons is responding. Rods are found in brains of mice overexpressing α-synuclein [312], a model for Parkinson’s disease with dementia (PDD) frequently occurring in patients with triplication of the α-synuclein gene [225]. In unreviewed work available online (bioRxiv 2021.02.02.425931; doi: 10.1101/2021.02.02.425931) Oliveira da Silva et al., showed that cofilin-actin rod pathology developed over a similar time span as coginitive deficits in an α-synuclein overexpressing mouse model.How do the various and structurally divergent rod inducers result in cofilin-actin rod formation in the same subpopulation of neurons? For Aβ alone, many binding partners have been identified [313], including lipids, ion channels, receptors, and the cellular prion protein (PrP^C^), which is a glycosyl-phosphatidyl-inositol (GPI)-linked protein on the membrane outer leaflet that accumulates in sphingolipid-enriched lipid rafts, sites of transmembrane receptor signaling and recycling [314]. A requirement for PrP^C^-expression for synaptic impairment and cognitive deficits in AD mouse models [315,316] triggered studies of PrP^C^ in rod formation. Neurons from PrP^C^-null mice do not form cofilin-actin rods in response to Aβ, proinflammatory cytokines, or gp120, but glutamate-induced rods were unaffected [281,310].

**Figure 10:**
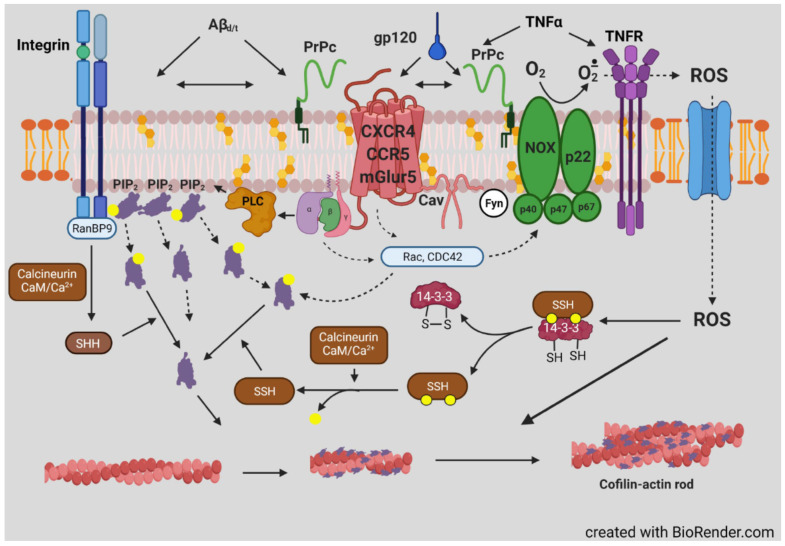
Proposed signaling for cofilin actin rod formation in neurons from multiple factors that have been implicated in dementia. Membrane lipid raft domains are enriched in sphingolipids, cholesterol, phosphatidylinositol phosphates [319], and PrP^C^ in the exoplasmic leaflet. Signaling to form cofilin-actin rods from Aβ, TNFα, and HIV gp120 requires PrPc, a functional NOX, and a G-protein coupled receptor (chemokine receptors CXCR4, CCR5, or mGluR5) presumably for stimulating PLC isoforms for hydrolysis of membrane PIP_2_ to release phospho-cofilin and dephospho-cofilin along with IP_3_ to increase cytoplasmic Ca^2+^. Activation of SSH1 by calcineurin [98] or via removal of 14-3-3 from SSH1L by oxidation [95] enhances the active cofilin pool, which can locally saturate F-actin, severing filaments into cofilin-saturated fragments, perhaps enhanced by Aip1. Pieces of cofilin-saturated F-actin can undergo ROS-induced oxidation to form intermolecular disulfide-linked cofilin dimers between fragments, ultimately bundling them into rods. Superoxide is released extracellularly from NOX, but ROS re-enters the cell via aquaporins [320]. Domains of PrPc may insert into the plasma membrane to interact with caveolin-1 and the non-receptor tyrosine kinase fyn for activating other downstream targets [321]. In this context, PrPc serves as more than just a membrane scaffold [322].

PrP^C^ is highly enriched in lipid rafts, sphingolipid, and cholesterol enriched membrane domains that differ from other regions of the plasma membrane in their composition and thickness. Many transmembrane receptors need to be translocated to these domains for either optimal extension of their transmembrane domains or interaction with cholesterol through their CARC/CRAC motifs [317]. Among these proteins are the family of NADPH oxidases (NOX), a ROS producing enzyme in which its activity increases in the early stages of AD [318]. NOX inhibition using both pharmacological and genetic methods eliminated rod induction from the protein inducers but not from glutamate. Rods formed in response to the PrP^C^-NOX pathway persist in neurites during continuous exposure but reverse in the presence of the inducer by inhibiting NOX, suggesting that continuous production of ROS is necessary for maintaining rods induced in this manner (Figure 10).

Surprisingly, neuronal overexpression of PrP^C^ induces rods in a higher percentage of neurons (>40%) than respond to Aβ or proinflammatory cytokines, but NOX is still required for the rod response [281], suggesting that the differences in PrP^C^ expression within neurons may modulate their rod response to the disease-associated rod-inducing factors. The GPCRs associated with PrPc rafts may function in rod formation through PLC to release cofilin for its local activation with IP_3_ serving to enhance Ca^2+^-release from intracellular stores to activate the calcineurin/SSH1 pathway and cofilin dephosphorylation [323].

Whether localized production of ROS is sufficient to induce rod formation was investigated using KillerFirefly, a genetically encoded light-sensitive ROS generating probe [324]. ROS was produced at sites of F-actin by targeting the probe with an actin-binding peptide. Cofilin-actin rods were formed within the region of ROS production, demonstrating local elevation of ROS in proximity to F-actin, which presumably has cofilin bound along some regions, can generate rods. Local ROS production induced rods followed in cells by a rod reporter named CofActor, which bridges cofilin to actin with a light responsive blue-light switch [119]. CofActor localizes to rods induced by either light or energetic/oxidative stress. Rod formation in HeLa cells in response to 2-deoxyglucose-mediated energy depletion (blocking glycolysis) occurs peripherally nearby sites of NOX-dependent ROS production, whereas inhibitors of mitochondrial ATP production, also releasing ROS, results in rods throughout the cytoplasm.

Multiple tropic forms of HIV exist in which different gp120 mutations have occurred, and these can differentially target two well studied GPCRs, CXCR4 (X4), and CCR5 (R5). These co-receptors serve roles in virus uptake into non-neuronal cells but until recently were thought to be expressed only on glia [311]. Specific antagonists of these receptors have gained FDA approval: the CCR5 antagonist maraviroc (Selzentry^®^) and the CXCR4 antagonist AMD3100 (Plerixafor^®^). In rodent neurons, X4 and R5 tropic forms of gp120 induced neuronal rods that were fully or partially inhibited by the specific antagonist of their known receptor. The X4 antagonist AMD3100 had the greatest inhibitory effect on rods induced by dual tropic gp120 [310]. Furthermore, rods induced by Aβd/t were similarly inhibited by both AMD3100 and maraviroc, suggesting that CXCR4 and CCR5 receptors are participants in the PrP^C^/NOX-mediated rod pathway. Since G-protein activation from these receptors can directly activate PLC [325], cofilin released from PIP_2_ on the raft cytoplasmic domain may serve to drive local rod formation (Figure 10).

Rod formation mediated by the gp120 receptors might explain many beneficial effects mediated by CXCR4 and CCR5 antagonists. In an acute mouse AD model initiated by brain injection of the pathological core of Aβ (Aβ_25–35_) or in traumatic brain injury (TBI) in which proinflammatory cytokines induce rod formation, the administration of CXCR4 and CCR5 antagonists improved recovery, ameliorated cognitive impairment, and reduced neuroinflammation [326,327,328]. Knock down of CCR5 in mice improved motor recovery after stroke and improved cognition after TBI; these improvements also occur in animals treated with maraviroc [329]. Maraviroc has been used in human subjects with HAND resulting in neurocognitive enhancement in the subjects receiving the drug over those in the placebo group [330]. Whether these improvements result from rod reduction by direct effects on neuronal receptors or work via their effects on microglia, a major source of released proinflammatory cytokines [331] is not yet known. However, a reduction in rods, if occurring, is only one of many possible ways in which these drugs could benefit neuronal function.

### 8.6. Rods and Tau Pathology

Preceding the development of tau pathology, changes in actin-dependent processes, including the formation of rods and mis-localization of Drp1, have been observed in neurons in different neurodegenerative disease models [224,267,312,332,333], suggesting that the sequestering of cofilin into cofilin-actin rods could be contributing to defects in many other diseases. We suggest that cofilin dysregulation also might be the initiator of tau pathology. Indeed, the displacement of tau from microtubules accompanying rod formation [42] or in response to cofilin hyperactivation [1] may be the initial step resulting in tau pathology. Many of the tau ser/pro or thr/pro pathological phosphorylation sites bracket tau’s microtubule binding interface, and each of the sites contributes in accumulating sufficient hyperphosphorylation to induce toxicity [334]. The sites are more accessible if tau is free from microtubules.

### 8.7. Neuronal Death

The final stage of all neurodegenerative diseases is neuronal death. Both necrosis and apoptosis contribute to neuronal demise in neurodegenerative diseases. Defects in mitochondrial metabolism, mitophagy, and mitochondrial transport all participate in some aspects of the death process (Figure 9). Cofilin KO has been shown to be protective against mitochondrial-induced apoptosis associated with ischemia [335]. In this regard, cofilin sequestration into rods may be beneficial in helping to sustain ATP levels by decreasing actin turnover [336]. However, loss of cofilin activity may also prevent actin reorganization associated with beneficial mitochondrial fission/fusion and mitophagy processes (Figure 9).

Phosphorylated cofilin is an activator of PLD1, which increases the levels of the E3 ubiquitin ligase MDM2, one target of which is the proapoptotic p53 protein resulting in its degradation [337]. Thus, one might expect that conditions that result in cofilin-actin rod formation also reduce pools of phospho-cofilin and, thus, should result in a larger pool of functional p53. However, rods form in neurites quite removed from the soma and their influence on the phospho-cofilin pool does not extend very far away from the rod region [281]. Although there might be some local effects on mitochondria through cofilin-p53 targeting, rod formation might have a greater effect in reducing PLD1 activity and increasing p53 pools for triggering cell death under ischemic conditions in neurons where rods are more plentiful and widely distributed than in age-related dementias.

## 9. Rod Modulatory Effects of Other Proteins

Due to the fact that cofilin and actin are critical to so many essential cell functions, genetic diseases from mutations in these proteins generally are found only in tissue specific isoforms, such as in muscle, where cofilin-2 is often the major or only expressed ADF/cofilin isoform and the only actin is the α-skeletal muscle isoform. However, with the plethora of associated proteins that cooperate or antagonize cofilin-mediated dynamics, it is worth considering how each of these proteins in neurons might impact rod formation by altering the levels of cofilin-saturated F-actin fragments used in rod assembly. For example, CAP2 is downregulated in AD and would, thus, not be readily available for depolymerization of cofilin-actin fragments, allowing them to accumulate. Indeed, in CAP2 knockout cortical neurons, there is a 35% increase in F-actin, a 50% decline in phosphorylated cofilin, and extensive cytoplasmic aggregates of cofilin [157]. We would expect proteins that contribute to cofilin inactivation by enhancing its phosphorylation or degradation to be rod inhibitory, while those enhancing the formation of cofilin-saturated stimulating ROS production would be rod-inducing (Figure 7).

## 10. Conclusions and Future Directions

Cofilin sequestration into rods may also impact synapse function by altering dendritic spine dynamics and synaptic vesicle release. ADF and cofilin seem to be able to replace each other in presynaptic and postsynaptic functions in mice, but ADF in the presynaptic compartment of excitatory synapses cycles actin assembly dynamics in response to depolarization more similarly to WT than what occurs in ADF-null mice expressing only cofilin [338]. Rodent models of AD, autism, manic/bipolar disorder, and sleep deprivation all show improved behavior/cognition through increasing cofilin phosphorylation [339]. However, the only definitive method of showing that rod formation is necessary for cognitive impairment is to generate a mouse in which cofilin-actin rods do not form. Two non-rod forming functional cofilin mutants, R21Q and K22Q, have been used to make rod reporters [289], but these two mutations are in the nuclear localization sequence, which could complicate interpretations of their use for studying a rod response. One other mutated form of cofilin, CC39,147AA, which cannot form the disulfide cross-link and, thus, does not incorporate into rods, seems to maintain its normal actin binding and depolymerizing functions [65]. Perhaps making a mouse encoding this mutant form, or even better a CC39,147SS mutant in which hydrogen bonding from these residues will be maintained, could help resolve this major question. Initial studies of such a mutant expressed in human iPSCs in which endogenous cofilin has been silenced and then converted to neurons could be beneficial in first determining if an inability to form rods protects against axonal transport defects or synapse loss in cultures treated with age-related neurodegenerative inducers, such as Aβ. The reintroduction of WT cofilin into these cells to restore rod formation could then be studied for its detrimental or beneficial effects. However, if the transient formation of cofilin-actin rods is required to preserve energy during stress or to modulate neurite outgrowth for purposes of pruning during brain development, animals expressing non-rod-forming mutants might have their own issues. At the very least, a neuronal specific knock-in of the non-rod forming mutant would be prudent if studies progressed to developing a mouse.

Although rods from energetically stressed cells have been isolated and characterized, components of rods that develop during neurodegenerative disease progression, other than actin and cofilin, are unknown. Since rod formation induced by the PrP^C^/NOX pathway is in a small subpopulation of neurons and fewer rods form per neuron than during energetic stress, their isolation for compositional analysis is challenging. One approach might be to use rod reporters to visualize the rod and laser capture microdissection coupled to mass spectrometry for their compositional analysis [340]. Certainly, much work lies ahead to obtain a complete understanding of the many beneficial and detrimental activities of this interesting ADF/cofilin protein family.
